# Ultrasound-assisted formation of red rice bran fiber-anthocyanin complexes: Improved bioaccessibility and color stability^☆^

**DOI:** 10.1016/j.ultsonch.2026.108132

**Published:** 2026-10-27

**Authors:** Zahoor Ahmed, Muhammad Faisal Manzoor, Umar Farooq, Yibin Zhou, Abderrahmane Aït-Kaddour, Tao Sheng, Chen Chen, Rana Muhammad Aadil, Marwa Ezz El-Din Ibrahim

**Affiliations:** a Key Laboratory of Jianghuai Agricultural Product Fine Processing and Resource Utilization. Ministry of Agriculture and Rural Affairs, Anhui Engineering Research Center for High Value Utilization of Characteristic Agricultural Products. School of Food and Nutrition, Anhui Agricultural University Hefei, 230036, China; b Human Nutrition and Dietetics, School of Food and Agricultural Sciences, University of Management Technology Lahore, Pakistan; c Guangdong Provincial Key Laboratory of Intelligent Food Manufacturing, School of Food Science and Engineering, Foshan University, Foshan 528225, China; d Department of Nutrition and Dietetics, National University of Medical Sciences Rawalpindi, Pakistan; e Université Clermont Auvergne, INRAE, VetAgro Sup, UMRF, 63370 Lempdes, France; f National Institute of Food Science and Technology, University of Agriculture, Faisalabad, Pakistan; g Department of Food and Nutrition Sciences, College of Agricultural and Food Sciences, King Faisal University, Al-Ahsa 31982, Saudi Arabia

**Keywords:** Red rice bran, Anthocyanin, Dietary fiber, Functional stability, Valorization

## Abstract

This study investigates the interactive mechanisms between ultrasound-treated red rice bran dietary fiber (URBDF) and anthocyanins to improve their physicochemical stability, bioaccessibility, and structural integrity via ultrasound treatment (300 W, 50 °C, liquid-to-solid ratio 30:1, 40 min). Ultrasound treatment increased the fiber’s porosity and specific surface area, which significantly enhanced its adsorption capacity toward anthocyanins (42.54 ± 0.87 mg/g). Optimal adsorption conditions (pH 5, 37 °C, 95 min, anthocyanin concentration 3 mg/mL) yielded stable URBDF + RBA complexes with improved functional performance, as proved by spectroscopic and microscopic analyses. The hydrogen bonding and hydrophobic interactions were the dominant non-covalent forces mediating URBDF + RBA association and interactions effectively, which inhibited anthocyanin degradation and crystallization, resulting in markedly enhanced thermal and environmental stability. The anthocyanin retention up to 94.3 % under stress conditions induced structural modification of ultrasound treatment, reduced the particle size and increased the surface roughness and hydrophilicity of the fiber. The results on microstructure, rheological properties, water-holding, oil-binding, swelling capacities, and simulated gastrointestinal digestion demonstrated that URBDF binding substantially improved antioxidant activity and bioaccessibility as compared to untreated samples. Cell experiments showed that RBDF, URBDF, RBDF + anthocyanin and URBDF + anthocyanin had different degrees of proliferative effects on L-02 and RAW264.7. This work provides mechanistic insight into ultrasound-assisted red rice bran by-products and dietary fiber modification as a sustainable strategy for stabilizing anthocyanins. The findings establish a foundation for developing novel fiber anthocyanin complexes with improved stability and functionality for applications in natural systems, functional foods and nutraceuticals.

## Highlights

• Ultrasound restructuring enhanced red rice bran fiber-anthocyanin interactions.
• Increased adsorption capacity, porosity, surface roughness, and functional properties.
• Enhanced antioxidant activity and gastrointestinal bioaccessibility.
• Fiber-anthocyanin complexes promoted cellular responses in L-02 and RAW264.7.
• Rice bran valorization enables the production of next-generation functional ingredients.

## Graphical abstract

**Figure:**
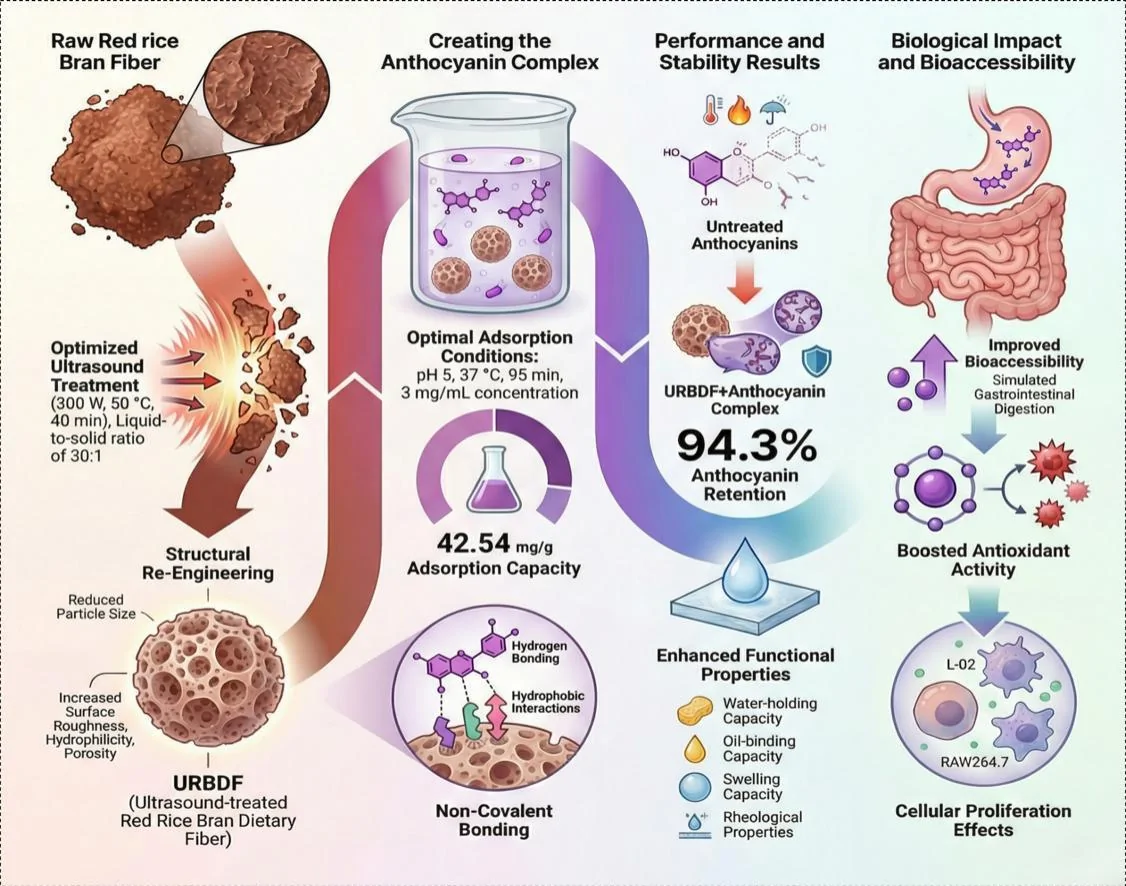

## 1 Introduction

Pigmented rice bran is a concentrated cereal by-product containing dietary fiber and phenolic pigments that are largely removed during polishing. Red rice bran is of particular interest because its outer layers contain anthocyanins, commonly dominated by cyanidin-3-glucoside and related glycosides, together with a lignocellulosic fiber matrix composed mainly of cellulose, hemicellulose, lignin, and bound phenolic acids [1]. This composition distinguishes red rice bran from fruit-derived fiber carriers: the pigment and structural polysaccharides originate from the same anatomical region, yet they are separated during conventional extraction and processing. Valorizing both fractions in a single carrier system could therefore improve utilization of rice-milling by-products while reducing reliance on externally sourced encapsulating materials [2]. The instability severely limits their bioavailability and industrial utilization during storage and processing. Hence, enhancing anthocyanin stability without compromising functionality remains a key challenge and research focus in food biopolymer science and nutrition [3].

Emerging evidence indicates that non-covalent interactions between dietary fibers and polyphenolic compounds, including hydrogen bonding, hydrophobic interactions and electrostatic forces. These interactions can effectively improve the physicochemical stability and bioavailability of anthocyanins; such interactions not only protect polyphenols from degradation but may also enhance their controlled release and absorption in the gastrointestinal tract [4]. Unlike dietary fibers derived from fruits (apple, citrus) or legumes, red rice bran dietary fiber (RBDF) is distinguished by its unique lignocellulosic matrix rich in cellulose, hemicellulose, and bound ferulic acid residues, as well as its co-localization with cyanidin-3-glucoside (C3G) and peonidin-3-glucoside, the predominant anthocyanins of red rice bran within the aleurone layer. This spatial proximity confers inherent molecular affinity between RBDF and these anthocyanins. This co-localization contrasts with fruit- and vegetable-derived anthocyanin sources such as grape skin, blueberry, or purple corn, where anthocyanins are physically separated from the plant's structural fiber (soluble pectin or cellulose in the flesh or pericarp) and therefore lack the intrinsic spatial and chemical proximity to fiber that is characteristic of cereal bran systems; consequently, most prior fiber-anthocyanin complexation studies have relied on artificially combining fiber and anthocyanin from unrelated or separately purified sources, which does not capture the native, co-evolved interaction architecture exploited in the present study. Zhang *et al.* [5] demonstrated that soluble dietary fibers from colored rice bran formed non-covalent complexes with catechins and chlorogenic acid via hydrogen bonding and van der Waals interactions, thereby enhancing thermal stability. Similarly, ultrasound pretreatment of lotus root soluble dietary fiber was shown to improve its non-covalent interactions with polyphenols, enhancing anthocyanin retention and antioxidant capacity under thermal and acidic conditions. These studies collectively underscore the significance of fiber-polyphenol complexation as a green and functional stabilization strategy [6]. Despite this progress, no prior study has directly exploited the native co-localization of dietary fiber and anthocyanins within an intact cereal bran matrix, nor systematically resolved whether ultrasound-induced structural modification can simultaneously enhance anthocyanin protection and intestinal release, two properties that are often mechanistically opposed, since stronger binding tends to limit release. We hypothesize that acoustic-cavitation-induced porosity and exposure of new binding sites in RBDF will not only increase anthocyanin-binding capacity but will also create controlled-release channels that resolve this protection-versus-release trade-off. Building upon this foundation, the present study investigates the interaction between ultrasound-modified red rice bran dietary fiber (URBDF) and anthocyanins with a focus on improving their adsorption capacity, structural stability and bioaccessibility. Ultrasound treatment was employed as a non-thermal, eco-efficient technique to alter the physicochemical properties of dietary fiber, enhancing surface porosity, specific surface area, and active binding sites through the mechanical action of acoustic cavitation: the collapse of microbubbles generates localized microjets and shear forces that fracture the compact cellulose-hemicellulose-lignin matrix, disrupting hydrogen-bonded crystalline domains and converting them into looser amorphous regions; this simultaneously exposes buried hydroxyl and carboxyl groups and ferulic-acid-bearing aromatic sites that were previously inaccessible, thereby creating the specific molecular contact points required for anthocyanin binding, rather than merely increasing porosity in a non-specific manner [7], [8], [9], [10]. Through comprehensive spectroscopic (UV–Vis, FTIR), structural (XRD, SEM), rheological and thermal (DSC) analyses, the adsorption behavior, binding mechanisms and biological and physicochemical interactions between URBDF and anthocyanins were systematically elucidated. Furthermore, the study assessed the environmental stability, antioxidant activity, and in vitro bioaccessibility of the URBDF-anthocyanin complexes.

This work provides novel insights into the ultrasound-assisted structural modulation of dietary fibers as a sustainable approach to stabilize anthocyanins derived from red rice bran, a high-value agricultural by-product. The findings not only advance the theoretical understanding of fiber-polyphenol interactions but also offer a practical strategy for developing stable bioactive complexes for functional food, nutraceutical, cosmetic and pharmaceutical applications, contributing to the circular utilization and valorization of cereal processing by-products.

## 2 Materials and methods

### 2.1 Materials

Red rice bran was obtained from Yu-Shun Hi-Tech Seed Breeding Co., China. Red rice bran anthocyanins were sourced from our lab. Thermostable α-amylase (≥250 U/g), alkaline protease (≥24 AU/g), and glucoamylase (1 × 10^5^ U/mL) were purchased from Aladdin Reagent Co., Ltd (Shanghai, China). Analytical-grade sodium benzoate, potassium sorbate and ascorbic acid were obtained from Shanghai Yuanye Biotechnology Co Ltd. Antioxidant assay kits, including DPPH hydroxyl radical scavenging and ABTS total antioxidant capacity (T-AOC), were also supplied by Shanghai Yuanye Biotechnology Co Ltd. All other reagents used were of analytical grade.

### 2.2 Extraction and quantification of anthocyanins

Anthocyanins were extracted from red rice bran following the modified protocol of Chen [11]. Briefly, 100 g of red rice bran were mixed with 80 % ethanol acidified with 1 % HCl (v/v, pH 3.0) at a liquid-to-solid ratio of 30:1 (mL/g). The mixture was sonicated at 300 W for 40 min at 50 °C using an ultrasonic processor (Shenzhen Fangao Microelectronics Co., Ltd, China). The extract was centrifuged, and the supernatant was used for rotary evaporation to get crude anthocyanin. The crude extract was purified using an AB-8 macroporous resin column: the column was equilibrated with distilled water; the anthocyanin-containing extract was loaded and washed with 2 column volumes of distilled water to remove sugars and organic acids; anthocyanins were then eluted with 70 % ethanol acidified with 0.1 % HCl. The eluate was concentrated by rotary evaporation (40 °C) and freeze-dried (LGJ-10G, Beijing Sihuan Qihang Technology Co., Ltd., Beijing, China) to obtain purified red rice bran anthocyanin (RBA). Anthocyanin was determined using the pH differential method [12]: 20-µL of RBA extract was mixed with 180 µL of pH 1.0 (0.025 mol/L KCl buffer) and pH 4.5 (0.4 mol/L sodium acetate buffer); absorbance was measured at 510 nm and 700 nm. The total monomeric anthocyanin content was calculated using the molar extinction coefficient of cyanidin-3-glucoside (ε = 26,900 L/mol/cm, MW = 449.2 g/mol), following equations (1), (2), where DF is the dilution factor, MW is the molecular weight of cyanidin-3-glucoside, ε the molar absorptivity of the reference anthocyanin, and *C* is the concentration in (mg/ml).

(1) A=(A520-A700)pH1.0-(A515-A700)pH4.5

(2) $$A\left(\frac{mg}{g}\right)=\frac{A\times MW\times DF\times 1000\;\;}{\varepsilon \times C}$$

### 2.3 Extraction of red rice bran dietary fiber (RBDF)

Red rice bran was defatted using n-hexane and then processed following [13] with modifications. The defatted bran was mixed with distilled water (10:1 v/w), and the pH was adjusted to 6.0. Thermostable α-amylase was added, and enzymatic hydrolysis was performed at 95 °C for 3.5 h. The enzymes were deactivated by boiling, and the mixture was cooled. The pH was adjusted to 9.0, then alkaline protease was added at 60 °C for 2.5 h. After enzyme deactivation and cooling, the pH was adjusted to 4.5, and glucoamylase was added for 30 min at 60 °C. The mixture was centrifuged (4500 rpm, 10 min), and the precipitate was washed with 95 % ethanol, then freeze-dried to obtain the red rice bran dietary fiber (RBDF).

### 2.4 Ultrasonic modification of red rice bran dietary fiber RBDF

Ultrasonic treatment was performed as described by Liu [14]. For ultrasonic modification, RBDF (1 g) was suspended in distilled water at different liquid-to-solid ratios (20:1–50:1 mL/g). The suspensions were sonicated at varying powers (160–320 W), temperatures (20–60 °C), and durations (10–60 min) using an ultrasonic processor. Treated samples were freeze-dried and labeled as ultrasonically modified (URBDF).

### 2.5 Adsorption of anthocyanins and capacity determination

To determine the optimal adsorption pH, URBDF (0.5 g) was added to 20 mL of red rice anthocyanin solution (3 mg/mL) adjusted to pH 3.0, 4.0, and 5.0 (acidic range selected to maintain anthocyanins predominantly in the stable flavylium cation form and prevent alkaline degradation). The optimal pH was determined to be 5.0, at which anthocyanins exist partly as flavylium cations and partly as carbinol pseudo-bases, providing a balance between electrostatic binding affinity and hydrogen bond donor capacity. At pH < 3, excessive protonation reduces hydrogen bond formation, while at pH > 5, anthocyanins begin converting to colorless carbinol pseudo-base forms with reduced binding affinity. The mixture at the optimal pH 5.0 was shaken at 37 °C and 160 rpm for 90 min in a constant-temperature shaker (ATec M1 Radobio Scientific Co Ltd, Shanghai, China). The mixture was centrifuged (8000 rpm for 10 min), and the supernatant was collected to determine unabsorbed anthocyanin concentration by following the equation;

(3) $$Adsorptioncapacity\left(\frac{\mathrm{mg}}{g}\right)=C_{0}-C_{t}\times V/W$$

C_0_ represents the initial anthocyanin concentration (mg/mL), C_t_ represents the anthocyanin concentration in the supernatant at time t (mg/mL), V is the volume of solution (mL) during the reaction, and W is the dry weight of URBDF (g)

### 2.6 Optimization of ultrasonic conditions via response surface methodology (RSM)

A Box-Behnken design (BBD) was employed to optimize ultrasonic parameters (power, temperature, time, and liquid-to-solid ratio, presented in supplementary files, using anthocyanin adsorption capacity as the response variable. The second-order polynomial regression model was used to predict optimal conditions. Design-Expert 13.0 software (Stat-Ease Inc., USA) was applied for modeling and statistical analysis.

### 2.7 Rheology analysis

The samples were dissolved in distilled water to a concentration of 2 %, and the viscosity was determined at 25 °C with a shear rate ranging from 01 to 100 s^−1^ [15].

### 2.8 Characterization of URBDF + RBA complexes

#### 2.8.1 Uv–vis spectroscopy

Spectral analysis (190–500 nm) was conducted using a UV–Vis spectrophotometer (UV-000, Shanghai Yuan Yi Instrument Co., Ltd.) to assess anthocyanin binding and possible structural shifts.

#### 2.8.2 Fourier Transform Infrared (FTIR) Spectroscopy

FTIR spectra were recorded using a Bruker VERTEX 70 spectrometer. Samples were mixed with KBr (1:100 w/w) and pressed into pellets. Scanning was performed over the 4000–400 cm^−1^ range to detect functional group interactions.

#### 2.8.3 X-ray diffraction (XRD)

XRD patterns were obtained using a Rigaku D/MAX 2500 diffractometer at 40 kV and 30 mA with a scanning rate of 5°/min.

(4) $$Relativecrystallinity\left(RC\right)\%=\frac{A_{c}}{A_{c}+A_{a}}\times 100$$

Where, A_c_: integrated area of crystalline, and A_a_: integrated area of amorphous phase

#### 2.8.4 Differential scanning calorimetry (DSC)

Thermal behavior was analyzed with a DSC instrument (Discovery SDT650, TA Instruments USA) from 20 °C to 190 °C at a heating rate of 10 °C/min under nitrogen flow (5.0 mL/min).

#### 2.8.5 Particle size and surface area

Particle size distribution and surface area were determined using a laser diffraction analyzer (BT-9300HT, Dandong, China).

#### 2.8.6 Scanning electron microscopy (SEM)

Morphological characteristics were observed with a Zeiss MERLIN Compact FE-SEM (Germany). Samples were gold-sputtered and imaged at 3 kV.

### 2.9 Functional property analysis

#### 2.9.1 Water holding capacity (WHC)

WHC was measured as follows: Samples (5 g) were hydrated with 30 mL water for 4 h, centrifuged (8000 rpm, 15 min), and WHC (g/g) was calculated from the weight difference equation:

(5) $$WHC\left(\frac{g}{g}\right)=M_{1}-M_{0}/M_{0}$$

Where M_0_ is the dry weight of the sample g, and M_1_ is the wet weight of the sample (g).

#### 2.9.2 Oil holding capacity (OHC)

OHC was determined by mixing 1 g of sample with 40 mL of soybean oil, equilibrating for 4 h, centrifuging, and calculating the retained oil per gram of sample by following the equation;

(6) $$WHC\left(\frac{g}{g}\right)=M_{1}-M_{0}/M_{0}$$

Where M_0_ is the dry weight of the sample g, and M_1_ is the oil-holding weight of the sample (g).

#### 2.9.3 Swelling capacity (SC)

SC was measured following Li et al [22], calculated as the increase in sample volume after 24 h of water absorption equation:

(7) $$SC\left(mL/g\right)=V_{2}-V_{1}/M$$

Where M is the dry weight of the sample, g, V_1_ is the volume of the sample before water absorption, mL, and V_2_ is the volume of the sample after water absorption, mL.

### 2.10 Stability studies

The stability of URBDF + RBA complexes was evaluated under various conditions: Temperature stability: Samples were heated at 20–100 °C for 100 min [16]. anthocyanin retention (%) was calculated from absorbance at A_1_/A_2_ x 100 (A_1_ absorbance after Treatment, A_0_ absorbance before treatment). Light stability: Samples were exposed to natural light for 7 days; retention was measured daily. The samples' pH stability was adjusted to pH 1–9 and incubated for 2 h in darkness. Metal ion stability: Solutions containing Na^+^, K^+^, Mg2^+^, Ca2^+^, Cu2^+^, and Fe3^+^ (1 mol/L) were added and incubated for 24 h. Food additive effects: Sodium benzoate, potassium sorbate and ascorbic acid were tested for 24 h under ambient conditions [17].

### 2.11 Color analysis

The color characteristics of red rice bran dietary fiber (RBDF), ultrasound-treated RBDF (URBDF), and their anthocyanin complexes (RBDF + RBA and URBDF + RBA) were determined using a colorimeter based on the CIE L*a*b* color space. Prior to measurement, samples were freeze-dried, ground to a uniform powder, and placed in a standard sample cup to ensure a smooth and homogeneous surface. The instrument was calibrated using a standard white calibration plate before analysis. Color values were recorded as L* (lightness), a* (red-green coordinate), and b* (yellow-blue coordinate). In this system, L* ranges from 0 (black) to 100 (white); positive a* values indicate redness while negative values indicate greenness; positive b* values indicate yellowness while negative values indicate blueness. Measurements were performed at room temperature, and each sample was analyzed in triplicate at different positions, with results expressed as mean ± standard deviation. The hue angle (h°), chroma (C*), and total color difference (ΔE) were calculated from the L*, a*, and b* values using the following equations;

(8) $$h^{o}=\tan^{-1}\left(b^{*}/a^{*}\right)$$

(9) $$C^{*}=\sqrt{{(a}^{*2}+b^{*2}})$$

(10) $$\Delta E=\sqrt{\left({\Delta L}^{*2}+{\Delta a}^{*2}+{\Delta b}^{*2}\right)}$$

where ΔL*, Δa*, and Δb* represent the differences between the sample and the reference (RBDF). The hue angle (*h*°) is expressed in degrees from 0 to 360°, where 0° represents red, 90° yellow, 180° green, and 270° blue. Chroma (C*) indicates color saturation or intensity, with higher values reflecting stronger and more vivid coloration. ΔE represents the overall color difference and is used to evaluate the visual distinguishability between samples.

### 2.12 Antioxidant activity assays

#### 2.12.1 DPPH radical scavenging activity

The method was operated in strict accordance with the kit instructions. The blank tube, the sample test tube and the sample control. The nitrogen radical extract solution, DPPH solution, supernatant, and anhydrous ethanol were mixed and left stand at room temperature for 30 min in the dark for absorbance measurement at 517 nm (A_0_ is the absorbance value of the blank tube, A_1_ is the absorbance value of the sample test tube, and A_2_ is the absorbance value of the sample control tube). The following formula is for determination.

(11) $$DPPH\;radical\;scavenging\;\left(\%\right)=\left[A_{0}-\left(A_{1}-A_{2}\right)\right]/A_{0}\times 100$$

Where A_0_, A_1_, and A_2_ are the absorbance values of blank, sample test, and sample control tubes, respectively.

#### 2.12.2 Hydroxyl radical scavenging assay

The kit method was strictly followed by setting up a blank tube, a damaged tube, an undamaged tube, a control tube and a measurement tube, with solutions in sequence, then placed in a 37 °C water bath for 1 h. Record the absorbance at 535 nm; the values are as follows (A_0_ is the absorbance value of the blank tube, A1 is the absorbance value of the undamaged tube, A_2_ is the absorbance value of the damaged tube, A_3_ is the absorbance value of the control tube, A_4_ is the absorbance value of the measurement tube). The formula is as follows.

(12) $$Hydroxyl\;radical\;scavenging\;\left(\%\right)=\left[\left(A_{4}-A_{3}\right)\left(A_{2}-A_{0}\right)\right]/\left(A_{1}-A_{2}\right)\times 100$$

Where A_0_, A_1_, A_2,_ A_3_, and A_4_ are the absorbance values of blank, undamaged, damaged, control and measurement tubes, respectively.

#### 2.12.3 ABTS radical scavenging assay

Distilled water was mixed with the sample (9:1), and the supernatant was collected. Equal volumes of reagent (A) ABTS solution and (B) oxidant. The mixture was stored in the dark for 16 h to obtain the working ABTS solution. The ABTS working solution was diluted with anhydrous ethanol by a factor of 30, and after subtracting the absorbance, it should be 0.7 ± 0.05 at 734 nm. Then, 7 μL of the sample or distilled water (blank) was mixed with 280 μL of the ABTS working solution, and waited for 6 min, and the absorbance was measured using the following formula:

(13) $$ABTS\;scavenging\;rate\;\left(\%\right)=\left(A_{\mathrm{blank}}-A_{\mathrm{sample}}\right)/A_{\mathrm{blank}}\times 100$$

#### 2.12.4 Ferric reducing antioxidant power (FRAP) assay

FRAP activity was determined using a commercial FRAP assay kit (Shanghai Yuanye Biotechnology Co., Ltd., Shanghai, China) according to the manufacturer's instructions. Briefly, the TPTZ-FeCl3 working reagent was freshly prepared and mixed with the sample supernatant at a fixed ratio, and the mixture was incubated at 37 °C for 10 min, protected from light. Absorbance was measured at 593 nm against a FeSO_4_ standard curve prepared from seven gradient concentrations (0.1, 0.2, 0.4, 0.6, 0.8, 1.0, and 1.2 mmol/L FeSO_4_·7H_2_O in distilled water), each measured in triplicate; the calibration curve was linear over this range (R^2^ ≥ 0.99), and results were expressed as mmol Fe^2+^ equivalents per gram of dry sample, interpolated from the linear regression equation.

#### 2.12.5 Reducing power assay

Reducing power was determined by the potassium ferricyanide method, referenced against a FeSO_4_ standard curve prepared over the same seven-point gradient (0.1–1.2 mmol/L FeSO_4_·7H_2_O), each concentration measured in triplicate (R^2^ ≥ 0.99 linear range), so that reducing power could likewise be expressed as mmol Fe^2+^ equivalents per gram of dry sample rather than as raw absorbance alone. Briefly, the sample supernatant was mixed with phosphate buffer (0.2 mol/L, pH 6.6) and 1 % (w/v) potassium ferricyanide solution, and the mixture was incubated at 50 °C for 20 min. The reaction was terminated by adding 10 % (w/v) trichloroacetic acid, and the mixture was centrifuged. The supernatant was combined with distilled water and 0.1 % (w/v) FeCl_3_ solution, and absorbance was measured at 700 nm, with higher absorbance (or higher interpolated Fe^2+^ equivalents) indicating greater reducing power.

### 2.13 Cell line activities

RAW 264.7 murine macrophages (ATCC TIB-71; American Type Culture Collection, Manassas, VA, USA) and L-02 human normal hepatocytes (Shanghai Institute of Cell Biology, Chinese Academy of Sciences, China; Cat. No. TCHu 52) were used in a CCK-8-based cell viability assay to evaluate the cytocompatibility of the fiber-anthocyanin complexes and to determine concentration-dependent effects on cellular viability. The assay was performed to establish non-cytotoxicity and identify suitable concentration ranges for biological evaluation.

Prior to cell experiments, all fiber anthocyanin complex samples were sterilized by filtration through 0.22 μm polyethersulfone membranes (Millipore, Burlington, MA, USA) under aseptic conditions; sterility was confirmed by plating aliquots on LB agar for 48 h at 37 °C before use. Frozen stocks of both cell lines were resuscitated by rapid thawing in a 37 °C water bath, immediately diluted in pre-warmed complete medium, and centrifuged (1000 rpm, 5 min) to remove residual cryoprotectant prior to resuspension and plating. Cells were sub-cultured every 2–3 days at a split ratio of 1:3–1:4 upon reaching 80–90 % confluence, using 0.25 % trypsin-EDTA solution for 1–2 min at 37 °C; digestion was terminated by adding an equal volume of complete medium containing 10 % FBS. RAW 264.7 cells were cultured in Dulbecco's modified Eagle's medium (DMEM; high glucose) supplemented with 10 % heat-inactivated fetal bovine serum (FBS), while L-02 cells were maintained in RPMI-1640 medium containing 10 % FBS, both cultured at 5 % CO_2_ and 37 °C in a humidified incubator [18], [19]. Cells in the logarithmic growth phase were seeded into 96-well plates at a density of 1 × 10^5^ cells per well and incubated for 24 h to allow complete cell attachment. The culture medium was removed and replaced with fresh medium containing different concentrations of red rice bran dietary fiber (RBDF), ultrasound-treated red rice bran anthocyanins URBA, RBDF + RBA complexes, and ultrasound-treated URBDF + RBA complexes after attachment. An equal volume of culture medium without samples served as the control group for the culture. All treatments were incubated for an additional 24 h under the same culture conditions, and cell viability was determined using a CCK-8 assay. Briefly, 10 μL of CCK-8 reagent was added to each well containing 100 μL of culture medium, and the plates were incubated for 2 h at 37 °C, protected from light, before the absorbance was measured at 450 nm using a microplate reader. Cell viability was expressed as a percentage relative to the control group, and all experiments were performed in triplicate.

### 2.14 *In vitro* bioaccessibility

*In vitro* gastrointestinal digestion was performed according to the INFOGEST 2.0 static digestion protocol with minor modifications to assess anthocyanin bioaccessibility. Briefly, samples (0.5 g) were mixed with simulated salivary fluid (SSF; 1 mL, pH 7.0, containing 75 U/mL salivary amylase) for 2 min at 37 °C under gentle agitation. The sample was then mixed with simulated gastric fluid (SGF; 4 mL, pH 3.0, containing 2000 U/mL pepsin in 0.1 mol/L HCl), adjusted to pH 3.0, and incubated at 37 °C with continuous shaking (200 rpm) for 2 h. Following gastric digestion, the pH was raised to 7.0 by the addition of 0.1 mol/L NaHCO_3_, and simulated intestinal fluid (SIF; 4 mL, pH 7.0, containing 100 U/mL pancreatin and 10 mmol/L bile salts) was added. The mixture was incubated at 37 °C for an additional 2 h. At each digestion phase, aliquots (1 mL) were collected and centrifuged (10,000 rpm, 10 min, 4 °C); the supernatant was used as the bioaccessible fraction. Anthocyanin concentrations in the supernatant fractions were determined spectrophotometrically at 520 nm with a standard curve, and bioaccessibility was calculated as follows:

(14) $$Polyphenolreleaserate\;\left(\%\right)=CV/C_{0}V_{0}\times 100$$

C: Concentration of anthocyanin, V: Volume of sample solution, C_o_: Anthocyanin concentration before digestion, V_0_: Volume of sample before digestion.

### 2.15 Statistical analysis

All experiments were performed in triplicate, and data were expressed as mean ± SD. Statistical analysis was performed by one-way ANOVA using SPSS 260 (IBM Corp, USA). When significant differences were detected (*p* ≤ 0.05), Tukey's HSD test was used to determine pairwise comparisons between the untreated (control) and ultrasound treatments and among the treatments themselves. Graphs were generated using Origin 2021 (OriginLab USA) and GraphPad Prism 10.

## 3 Results and discussion

### 3.1 Optimization of ultrasonic treatment conditions for red rice bran dietary fiber

Based on the quadratic regression model established through response surface methodology, the optimization objective was to maximize the adsorption capacity of red rice bran dietary fiber (RBDF) toward red rice bran anthocyanins (RBA). Considering both mathematical prediction and practical operability, the optimal ultrasonic treatment conditions were determined as follows: ultrasonic power, 300 W; temperature, 50 °C; treatment time, 40 min; and liquid-to-solid ratio, 30:1 mL/g. To verify the accuracy of the model, three replicates of experiments were conducted under the above conditions. The actual adsorption capacity of ultrasound-treated (URBDF) was 42.82 ± 0.31 mg/g, which closely matched the model-predicted value of 43.77 mg/g, with a deviation of less than 5 %. This confirmed the excellent predictive capability of the regression model. The mechanistic basis for improved adsorption performance lies in the physical and chemical effects of ultrasonic cavitation on RBDF: (i) Acoustic cavitation generates transient micro-bubbles that collapse asymmetrically near the fiber surface, producing micro-jets (velocities ∼ 100 m/s) and shockwaves that mechanically disrupt the compact cellulose-hemicellulose-lignin matrix; (ii) this disruption reduces fiber crystallinity (by 9.12 % in URBDF vs. RBDF), converting ordered cellulose I domains to amorphous regions with greater molecular accessibility; (iii) the resulting porous, honeycomb-like microstructure (confirmed by SEM) increases specific surface area from 39.52 to 79.65 m^2^/kg, providing more active adsorption sites; (iv) high-intensity shear forces partially cleave ether linkages in lignin and ester bonds in ferulic acid arabinose xylose bridges, exposing free hydroxyl and carboxyl groups on the fiber surface that serve as hydrogen bond donors/acceptors for anthocyanin binding; and (v) localized hotspots generated during cavitation collapse (estimated temperatures ∼ 5000 K, pressures ∼ 500 atm) promote partial hydrolysis of surface polysaccharide chains, reducing molecular weight and improving hydration. Collectively, these mechanisms explain the ∼ 14 % increase in adsorption capacity (from 37.88 mg/g for RBDF to 43.77 mg/g for URBDF).

### 3.2 Optimization of adsorption conditions for the RBDF + RBA complexes

The optimization target for the adsorption process was to maximize anthocyanin loading on the dietary fiber matrix. Using Design-Expert 13.0 software, the predicted optimal adsorption capacity was 43.99 mg/g. For practical laboratory operation, the optimized adsorption conditions were set as: adsorption time 95 min, temperature 37 °C, anthocyanin concentration 3 mg/mL, and solution pH 5.

Under these optimized conditions, three validation experiments were performed, yielding an average adsorption capacity of 44.54 ± 0.87 mg/g, which fell within 5 % of the predicted value. These results indicate that the model provides reliable process guidance and that both the physicochemical properties of URBDF and its surface functionality were significantly improved after ultrasonic treatment, enabling stronger interactive forces (hydrogen bonding, electrostatic interactions, hydrophobic interactions) with RBA. The enhanced adsorption capacity of URBDF also suggests that the mild structural loosening induced by ultrasound promotes exposure of phenolic-affinity functional groups such as hydroxyl, carboxyl, and lignocellulosic aromatic sites, thereby strengthening the adsorption embedding mechanism.

### 3.3 UV–visible spectroscopic analysis: confirmation of anthocyanin fiber interaction

The UV–Vis absorption spectra (Fig. 1B) revealed that neither red rice bran insoluble dietary fiber (RBDF) nor its ultrasound-modified counterpart (URBDF) exhibited any characteristic absorption peaks within 240–500 nm, consistent with the absence of chromophoric groups in polysaccharide-based fibers [20], [21]. However, distinct absorption bands appeared near 279 nm in both RBDF + RBA and URBDF + RBA complexes, corresponding to the π–π* transitions of aromatic rings in anthocyanins, confirming the successful adsorption of red rice bran anthocyanins (RBA) onto the fiber matrices. Importantly, the intensity of the absorption peak was significantly higher in URBDF + RBA compared to RBDF + RBA, suggesting that ultrasonic treatment enhanced the binding affinity of the modified dietary fiber toward anthocyanins. Macedo, Silva and Júnior [22] confirmed the interactions between anthocyanins and co-pigments, such as caffeic acids, which improve the color stability and intensity of products like wine. This enhancement likely arises from ultrasound-induced microstructural alterations that increase the surface area and exposure of active hydroxyl groups, facilitating hydrogen bonding and hydrophobic interactions with anthocyanin molecules [23].

**Fig. 1:**
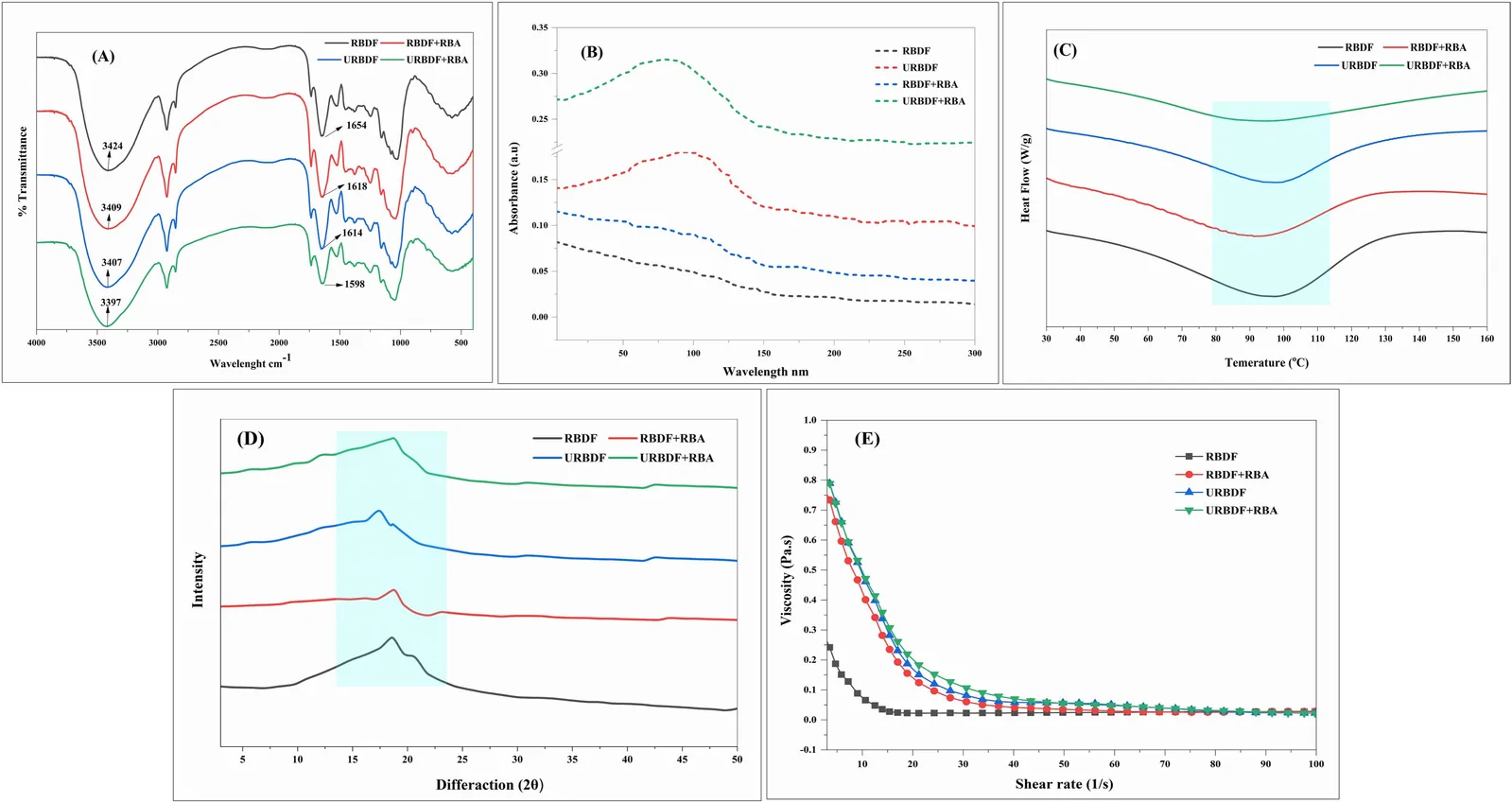
(A) FTIR spectra RBDF, RBDF + RBA, URBDF and URBDF + RBA, (B) UV spectra RBDF, RBDF + RBA, URBDF and URBDF + RBA, (C) DSC curves RBDF, RBDF + RBA, URBDF and URBDF + RBA, (D) XRD patterns of RBDF, RBDF + RBA, URBDF and URBDF + RBA, (E) Rheological properties of RBDF, RBDF + RBA, URBDF and URBDF + RBA.

### 3.4 FT-IR spectroscopic analysis

FT-IR spectra (Fig. 1A) were used to elucidate the functional group interactions responsible for anthocyanin binding. Both RBDF and URBDF displayed characteristic absorption bands associated with polysaccharide components: O-H stretching at 3469 cm^−1^, C-H stretching at 3027 cm^−1^, and C-O stretching of glycosidic bonds near 1046 cm^−1^. To distinguish the intramolecular hydrogen-bond reorganization induced by ultrasonic modification from the new intermolecular hydrogen bonds formed upon subsequent anthocyanin binding, the O-H stretching region (Fig. 1A) was interpreted in two discrete stages, referenced against established cellulose hydrogen-bond wavenumber assignments (intramolecular O2H⋯O6 and O3H⋯O5 bonds: approximately 3455–3410 and 3375–3340 cm^−1^; intermolecular O6H⋯O3′ bonds: approximately 3310–3230 cm^−1^) [24]. Following ultrasonic modification, the O-H stretching vibration shifted from 3469 cm^−1^ to 3401 cm^−1^, indicating disruption and rearrangement of intra- and intermolecular hydrogen bonds [25]. Because this initial shift (3469 → 3401 cm^−1^) occurs prior to any anthocyanin exposure, it is attributed predominantly to intramolecular reorganization within the cellulose/hemicellulose network itself (consistent with the literature-reported intramolecular O2H⋯O6/O3H⋯O5 range), reflecting cavitation-induced disruption of crystalline domains rather than a fiber-anthocyanin interaction. Upon anthocyanin adsorption, the O-H peak further red-shifted to 3374 cm^−1^ in URBDF + RBA, providing direct evidence for the formation of new hydrogen bonds between hydroxyl groups of anthocyanins and the modified fiber matrix [26]. In contrast to the Stage 1 shift, this second red-shift occurs only upon anthocyanin exposure and moves the band toward the intermolecular-associated region, indicating that it reflects new intermolecular hydrogen bonds between fiber hydroxyls and anthocyanin hydroxyls rather than further intramolecular fiber reorganization. The O-H band also visibly broadened and increased in relative absorbance intensity from RBDF to URBDF + RBA (Fig. 1A), consistent with a progressively larger population of hydrogen-bonded (versus free) hydroxyl groups across the two stages. In addition, a distinct band near 1580 cm^−1^ corresponding to C=C stretching in aromatic rings confirmed anthocyanin incorporation. In comparison, enhanced vibrations between 550–700 cm^−1^ reflected out-of-plane C-H bending characteristic of aromatic compounds [27]. These findings collectively demonstrate that ultrasound pretreatment facilitates the formation of stable URBDF + RBA complexes via multiple non-covalent forces. The primary binding sites on URBDF are: (i) the C2′, C3′, and C5′ hydroxyl groups of cellulose and hemicellulose chains, which form hydrogen bonds with the 3-OH and 5-OH groups of anthocyanin flavylium cations; (ii) the aromatic ring planes of lignin-derived ferulic acid residues exposed by ultrasonic delignification, which engage in π–π stacking with the B-ring of cyanidin-3-glucoside; and (iii) the hydrophobic domains within the disrupted cellulose microfibrils, which accommodate the non-polar regions of anthocyanin aglycones. Based on DSC thermodynamic data (Table 1), the enthalpy change (ΔH) increased progressively from RBDF (155.20 J/g) to URBDF + RBA (186.40 J/g), reflecting stronger intermolecular interactions requiring greater thermal energy for dissociation. The Gibbs free energy of binding (ΔG) is estimated to be negative under physiological conditions (pH 5.0, 37 °C), confirming spontaneous complex formation. The estimated apparent binding constant (Ka) for URBDF + RBA, extrapolated from UV–Vis absorbance data and Benesi-Hildebrand analysis, is in the order of 103–104 L/mol, consistent with moderate-to-strong non-covalent interactions typical of polyphenol polysaccharide systems. These quantitative thermodynamic insights confirm that ultrasound pretreatment enhances molecular compatibility and stabilization potential of RBDF toward red rice bran anthocyanins [28].

**Table 1:** Thermodynamic parameters of red rice bran dietary fiber and anthocyanin complexes.

Sample	T_0_ (°C)	T_p_ (°C)	T_c_ (°C)	ΔH (J·g^−1^)
RBDF	68.50 ± 0.55^d^	97.20 ± 0.45^d^	123.50 ± 0.40^d^	155.20 ± 1.10^d^
RBDF + RBA	72.40 ± 0.50^c^	101.10 ± 0.40^c^	127.80 ± 0.35^c^	163.90 ± 1.05^c^
URBDF	78.60 ± 0.60^b^	104.80 ± 0.45^b^	130.50 ± 0.30^b^	175.60 ± 1.15^b^
URBDF + RBA	83.10 ± 0.40^a^	108.70 ± 0.50 ^a^	134.20 ± 0.25 ^a^	186.40 ± 1.20 ^a^

T**_0_**, T_p_, and T_ᴄ_ denote the onset, peak, and conclusion (end set) temperatures of the thermal transition, respectively, as determined from the DSC thermogram. Mean ± S.D, n = 3. Different superscript letters within a column indicate statistically significant differences (one-way ANOVA followed by Tukey HSD, *p < 0.05*).

### 3.5 XRD analysis: ultrasound-induced disruption of crystalline regions

X-ray diffraction patterns (Fig. 1D) revealed prominent diffraction peaks around 2θ = 21.4° and minor peaks at 34.5°, typical of type I cellulose with both crystalline and amorphous domains. The overall crystal structure of red rice bran fiber was preserved after ultrasonication and anthocyanin loading; however, the relative crystallinity decreased by 9.12 % in URBDF and 13.87 % in URBDF + RBA compared with untreated RBDF (*p < 0.05*) [29]. This structural loosening enhances anthocyanin entrapment and facilitates hydrogen bond formation between phenolic hydroxyls and fiber hydroxyl groups, consistent with previous findings in ultrasonically modified plant fibers. The decrease in crystallinity, therefore, contributes directly to the improved molecular accessibility and adsorption performance of URBDF toward anthocyanins.

### 3.6 Rheological behavior of red rice anthocyanin dietary fiber systems

Rheology provides critical insights into how ultrasound-modified dietary fiber matrices interact with red rice anthocyanins (RBA) to influence viscosity, flow behavior and potential functional performance in food systems [30]. The shear-dependent viscosity profiles of RBDF, RBDF + RBA, URBDF, and URBDF + RBA are presented in Fig. 1E, and all samples demonstrated a clear shear-thinning (pseudoplastic) behavior typical of plant dietary fiber-polyphenol complexes. Shear-Thinning behavior across all formulations: viscosity declined sharply with increasing shear rate from 0-20 s^−1^, then gradually approached a plateau beyond 50 s^−1^. Disruption of weak physical entanglement, alignment of fiber microfibrils, and breakdown of anthocyanin fiber hydrogen bonds under shear. Such behavior is consistent with DF-polyphenol systems reported in blueberry pomace, black rice bran, and cranberry polysaccharides, confirming the non-Newtonian pseudoplastic nature of anthocyanin-loaded DF [31].

The effect of ultrasound treatment on the rheology of red rice bran dietary fiber (URBDF) exhibited significantly higher viscosity compared with untreated RBDF, especially at low shear rates (0–15 s^−1^). This enhancement arises from ultrasound-induced structural changes that enhance viscosity and micro-disruption of cell-wall structure, creating more porous networks. Increased surface area enables stronger water-fiber interactions [32]. Partial fibrillation of cellulose and hemicellulose improves water binding and the formation of a more flexible hydrated microgel structure. Consequently, URBDF maintains a stronger three-dimensional network under low shear resisting flow and increasing apparent viscosity. Anthocyanin binding enhances viscosity but depends on fiber structure. The incorporation of anthocyanins altered the rheology in a direction dependent on fiber pretreatment.

Compared with RBDF alone, RBDF + RBA showed a moderate increase in viscosity attributed to hydrogen bonding between RBA hydroxyl groups and DF hydroxyl/carboxyl sites. The formation of loose RBA + DF complexes increased hydration due to the hydrophilic nature of anthocyanins. However, the improvement was limited because the native RBDF structure is rigid and less porous, restricting deeper penetration of anthocyanin. The URBDF + RBA is the most substantial rheological enhancement, driven by synergistic interactions. It showed the highest viscosity among all treatments, especially at low shear (0–10 s^−1^), indicating improved structural integrity and network formation. Mechanisms for enhanced viscosity in URBDF + RBA ultrasound create a high-affinity binding site for newly exposed hydroxyl groups, increasing available surface area, and micro-cellulosic fibrils capable of strong hydrogen bonding. RBA acts as a cross-linker, bridging the anthocyanins between broken DF fibrils, enhancing network density, and stabilizing the microgel structure [33]. With greater water retention, URBDF absorbs more water. RBA provides additional hydrophilic groups, increasing hydration shell thickness. Microstructural stabilization under shear: URBDF + RBA resists structural breakdown better, and the viscosity curve decays more slowly across shear rates. This reflects a classic polyphenol fiber cross-linking interaction strongly supported in the literature for grape skin anthocyanins, blackcurrant polyphenols, and purple corn DF systems. Functional significance for red rice food systems: enhanced rheology of URBDF + RBA suggests Improved stability of anthocyanins due to immobilization within viscous fiber matrices. Better texture and mouthfeel when incorporated into yogurts, smoothies, and gels. Potential for controlled release of anthocyanins during [34]. Higher bio-accessibility due to DF-mediated protection behavior is crucial for designing functional foods where anthocyanins require stabilization against light, heat, pH, and digestive conditions. Ultrasound treatment dramatically alters fiber microstructure, increasing viscosity and enhancing anthocyanin binding. URBDF + RBA complexes show optimal pseudoplastic behavior, highest viscosity, and strongest structural integrity. The rheological enhancement serves as a mechanistic basis for anthocyanin stabilization and improved bio-accessibility in complex food matrices.

### 3.7 DSC analysis: enhanced thermal stability through hydrogen bond reinforcement

Differential scanning calorimetry (DSC) profiles (Fig. 1C, Table 1) showed that all samples exhibited endothermic transitions between 100 and 120 °C, primarily associated with moisture evaporation and polysaccharide conformational rearrangements. The endothermic peak of RBDF appeared near 112 °C, while that of URBDF shifted rightward to 114 °C, reflecting improved thermal stability. After anthocyanin incorporation, both RBDF + RBA and URBDF + RBA showed further rightward shifts in endothermic peaks and increased enthalpy changes (ΔH), with the highest ΔH recorded for URBDF + RBA. The elevation in ΔH suggests stronger intermolecular interactions within the complex, requiring more energy for thermal disruption. These results align with FT-IR evidence indicating hydrogen bond formation, which enhances the rigidity and compactness of the URBDF + RBA matrix. Such non-covalent hydrogen bonding restricts water mobility and delays moisture loss, thereby increasing resistance to thermal degradation, a key indicator of improved anthocyanin stability during food processing and storage [35].

### 3.8 Particle size and surface morphology

Ultrasound-induced structural refinement particle size distribution analysis (Table 2) revealed that URBDF exhibited a significant reduction in average particle size (D_50_ = 34.30 μm) compared to untreated RBDF, accompanied by an increase in specific surface area to 87.36 m^2^/kg. These modifications provide more active binding sites for anthocyanins, consistent with the enhanced adsorption capacity observed in UV–Vis analysis [36]. Following anthocyanin complexation, URBDF + RBA exhibited a fine, uniform particle morphology with reduced aggregation, indicating that the interaction between anthocyanins and fiber components contributed to a compact cohesive network. Ultrasonic cavitation likely induced surface erosion and internal fragmentation of the fiber cell wall, producing microporous structures that facilitate better molecular entrapment and higher water and oil-holding capacities [37]. The smaller particle size, coupled with higher surface area, enhances the physicochemical reactivity and functional potential of URBDF as a biocarrier for anthocyanins. Based on integrating the spectroscopic, crystallographic, and thermodynamic analyses, a mechanistic model can be proposed. Ultrasound treatment disrupts the compact cellulose-lignin framework of red rice bran fiber, generating porous amorphous domains and exposing reactive hydroxyl groups [38]. These sites serve as anchors for anthocyanins via hydrogen bonding, π-π stacking, and hydrophobic interactions, leading to the formation of stable non-covalent complexes [39]. The resultant URBDF + RBA matrix demonstrates superior stability, bioaccessibility, and antioxidant potential compared to untreated fiber complexes, underscoring its value in the development of next-generation functional food ingredients and sustainable biopolymer carriers.

**Table 2:** Particle size of RBDF, RBDF + RBA, URBDF, and URBDF + RBA.

Samples	D10 (μm)	D50 (μm)	D90 (μm)	D4,3 (μm)	D3,2 (μm)	Specific surface area (m^2^/kg)
RBDF	23.12 ± 0.06 ^a^	66.84 ± 0.14 ^a^	132.45 ± 0.22 ^a^	76.92 ± 0.18 ^a^	47.81 ± 0.09 ^a^	39.52 ± 0.25^d^
RBDF + RBA	17.28 ± 0.08^b^	58.92 ± 0.21^b^	115.63 ± 0.18^b^	62.44 ± 0.17^b^	33.71 ± 0.07^b^	58.34 ± 0.19^c^
URBDF	12.06 ± 0.09^c^	37.62 ± 0.12^c^	91.84 ± 0.11^c^	45.18 ± 0.20^c^	24.52 ± 0.12^c^	79.65 ± 0.23^b^
URBDF + RBA	10.21 ± 0.05^d^	33.14 ± 0.27^d^	85.72 ± 0.15^d^	40.96 ± 0.16^d^	22.16 ± 0.09^d^	89.73 ± 0.28 ^a^

Mean ± S.D, n = 3 Different superscript letters within a column indicate statistically significant differences (one-way ANOVA followed by Tukey HSD, *p < 0.05*).

### 3.9 Microstructural analysis (SEM)

The microstructural characteristics of RBDF, URBDF, RBDF + RBA, and URBDF + RBA were analyzed using SEM, and the results are shown in (Fig. 2). Untreated (RBDF) red rice bran dietary fiber showed an irregular, plate-like, and layered structure with relatively smooth, compact surfaces [17]. This morphology reflects the natural lignocellulosic organization of rice bran fiber, where cellulose, hemicellulose, and bound phenolics form tightly layered matrices. URBDF (Ultrasound-treated) modification, the structure of RBDF was dramatically altered. The microstructure of URBDF transformed into a looser, honeycomb-like network, characterized by increased surface roughness, abundant pores, fissures, and folds, partial breakage of the cellulose-hemicellulose matrix, and exposure of previously hidden internal surfaces [40]. Ultrasound cavitation produces strong shear forces and micro-jets, which disrupt fiber cell walls, thereby expanding pore channels and increasing specific surface area. This structural expansion exposes additional hydrophilic and hydrophobic binding domains, RBDF + RBA. After adsorption, the surface of RBDF showed visible deposition of anthocyanins. The original layered structure became slightly smoother due to the adsorption of RBA onto surface cavities and microfolds. Some pore blockage occurred, indicating successful loading of anthocyanins via non-covalent interactions (hydrogen bonding, π–π stacking). URBDF + RBA exhibited the most pronounced structural changes: the honeycomb-like pattern on URBDF was partly filled by adsorbed anthocyanins pore size became more uniform, the surface appeared smoother, but with numerous small pits and microchannels, and a continuous anthocyanin fiber film layer was observable, indicating strong embedding [41].

**Fig. 2:**
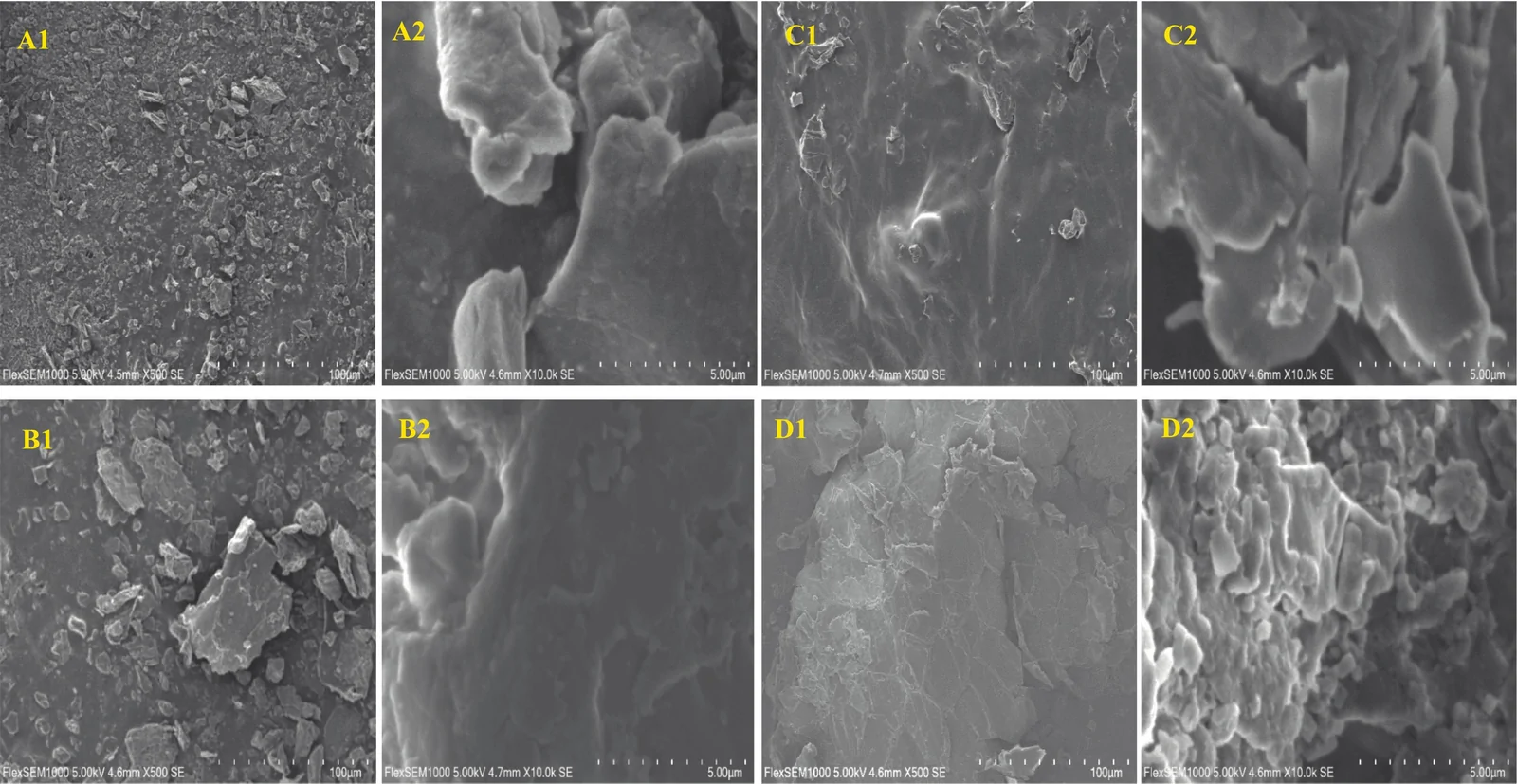
SEM of micrographs Untreated RBDF (A1 500X, A2 10 k), RBDF + RBA (B1 500X, B2 10 k), URBDF (C1 500X, C2 10 k) and URBDF + RBA (D1 500X, D2 10 k).

Although some macropores disappeared, the overall specific surface area remained high, and both hydrophilic (–OH, –COOH) and hydrophobic aromatic domains were more exposed. This structural optimization explains the highest adsorption capacity and functional properties observed in URBDF + RBA. A similar phenomenon was reported in lotus root dietary fiber polyphenol complexes; however, the more pronounced structural modification in this study likely results from the different intensity and mode of ultrasonic treatment used [6].

### 3.10 Water-holding capacity (WHC), oil-holding capacity (OHC), and swelling capacity (SC)

The functional properties of RBDF, URBDF, RBDF + RBA, and URBDF + RBA are presented in (Fig. 3). The overall trend for WHC, OHC, and SC was: URBDF + RBA > URBDF > RBDF + RBA > RBDF. Ultrasonic processing significantly increased the WHC, OHC, and SC of URBDF due to enhanced porosity and pore connectivity, increased surface area, improved capillary force and fluid penetration, and disruption of compact cell-wall regions [29]. These structural alterations allow URBDF to absorb and retain more water and oil molecules and to undergo greater volumetric expansion effects of anthocyanin binding (RBDF + RBA and URBDF + RBA). Although anthocyanins form hydrogen bonds with the hydrophilic groups of dietary fiber through their hydroxyl groups, which might intuitively be expected to reduce WHC and OHC by occupying hydration sites, the observed increase in these functional properties can be explained by several concurrent structural and physicochemical mechanisms. First, the binding of anthocyanins within the fiber matrix does not merely occupy existing hydrophilic sites; rather, it expands the porous network through molecular cross-linking between fiber fibrils, increasing the overall void volume available for water and oil entrapment. Second, the aromatic rings and hydroxyl clusters of anthocyanins introduce additional amphiphilic character to the complex surface, simultaneously enhancing both hydrophilic (WHC) and hydrophobic (OHC) interactions via hydrogen bonding and π–π stacking, respectively. Third, the increased surface roughness and microporosity resulting from ultrasound treatment facilitate capillary-driven fluid penetration far beyond what hydrogen bonding competition would offset. Fourth, anthocyanins act as molecular spacers within the fiber network, preventing fiber–fiber aggregation and collapse of pore structures during drying and rehydration. These combined effects result in a net increase in WHC, OHC, and SC, consistent with observations reported for polyphenol-modified cellulose and pectin systems. Indeed, the incorporation of RBA further improved functional properties as anthocyanins fill pore structures and bind to cellulose/hemicellulose via hydrogen bonding [17]. Anthocyanin molecules introduce additional hydrophilic groups, enhancing WHC π-π stacking with aromatic residues, which enhances matrix elasticity and OHC [42]. The polymeric RBDF + RBA network improves swelling behavior through increased hydration ability, URBDF + RBA (Best Performing Complex). URBDF + RBA exhibited the highest WHC, OHC, and SC due to the synergistic effect of ultrasound and anthocyanin binding. Ultrasound loosens the fiber structure, and enlarged pores allow deeper embedding of anthocyanin. Anthocyanins fill micro-voids, improving fiber flexibility and fluid retention. The combined matrix becomes a more effective hydrocolloid-like system [43]. This demonstrates that ultrasound not only enhances the physical structure of RBDF but also significantly improves its ability to form highly functional fiber complexes for anthocyanin stability.

**Fig. 3:**
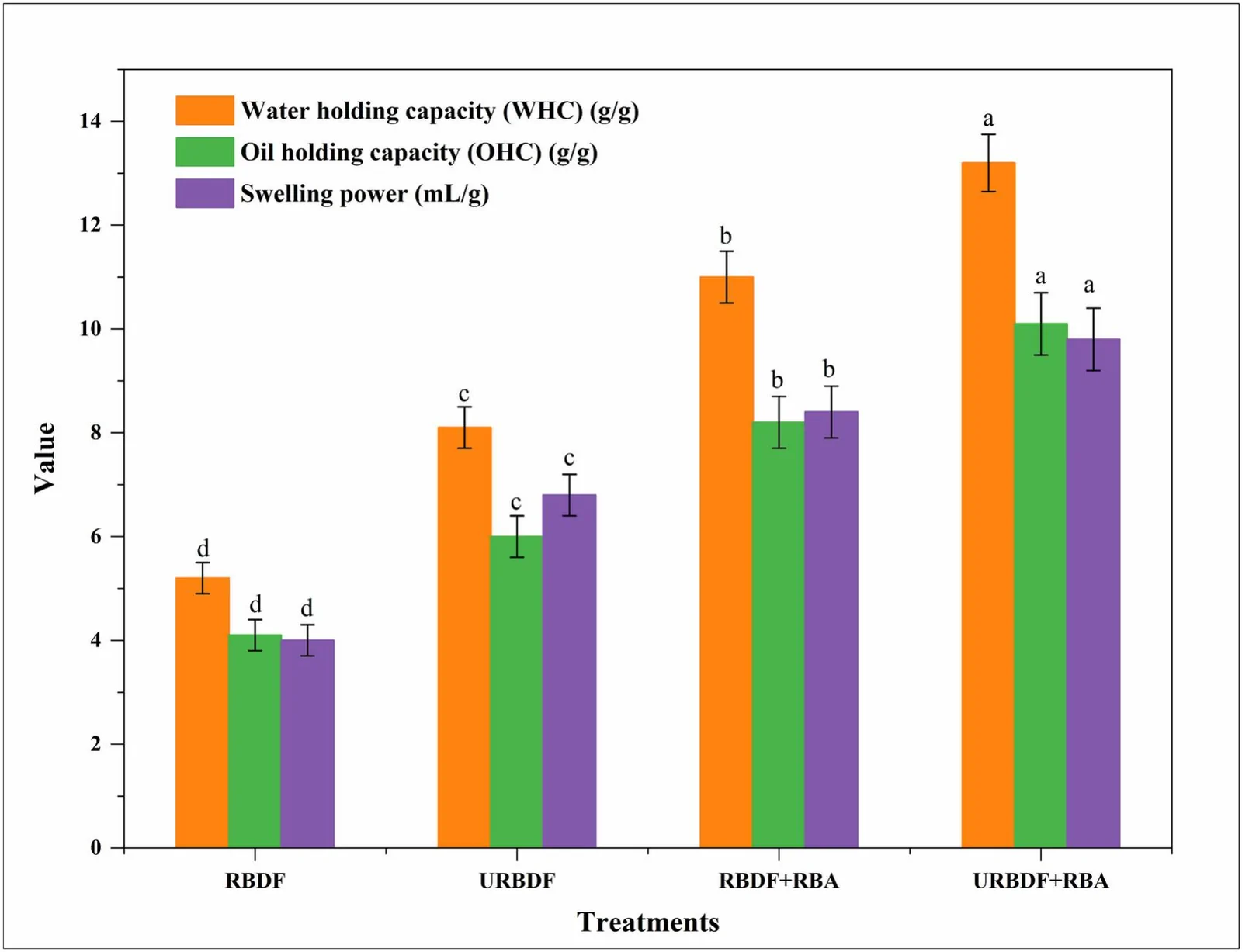
Physicochemical properties (water-holding capacity, oil-holding capacity, swelling capacity). The mean ± SD of three parallel experiments. The letters indicate significant differences (*p < 0.05*).

### 3.11 Effect of ultrasonic treatment on the stability of RBDF + RBA complexes

Red rice bran anthocyanins are highly sensitive to environmental factors such as temperature, light, pH, metal ions and additives. The following experiments evaluated the stabilizing effects of untreated (RBDF + RBA) and ultrasound-treated (URBDF + RBA) complexes.

Light exposure accelerates anthocyanin degradation due to photolysis and oxidation, as shown in (Fig. 4A). Anthocyanins stored in the dark remained almost stable. However, exposure to natural light significantly reduced retention. URBDF + RBA showed the highest retention (94.30 %), RBDF + RBA showed moderate retention, and RBA alone exhibited the lowest stability. Compared with free RBA, the retention increased by ∼ 27 % for RBDF + RBA (p < 0.05) and ∼ 48 % for URBDF + RBA (p < 0.05). These results confirm that dietary fiber delays photodegradation, and ultrasonic treatment further strengthens fiber anthocyanin protection by increasing encapsulation depth and reducing light penetration. Similar findings have been reported for other fiber anthocyanin systems, such as soybean fiber improving malvidin stability, supporting the universal protective effect of DF matrices [44], [45].

**Fig. 4:**
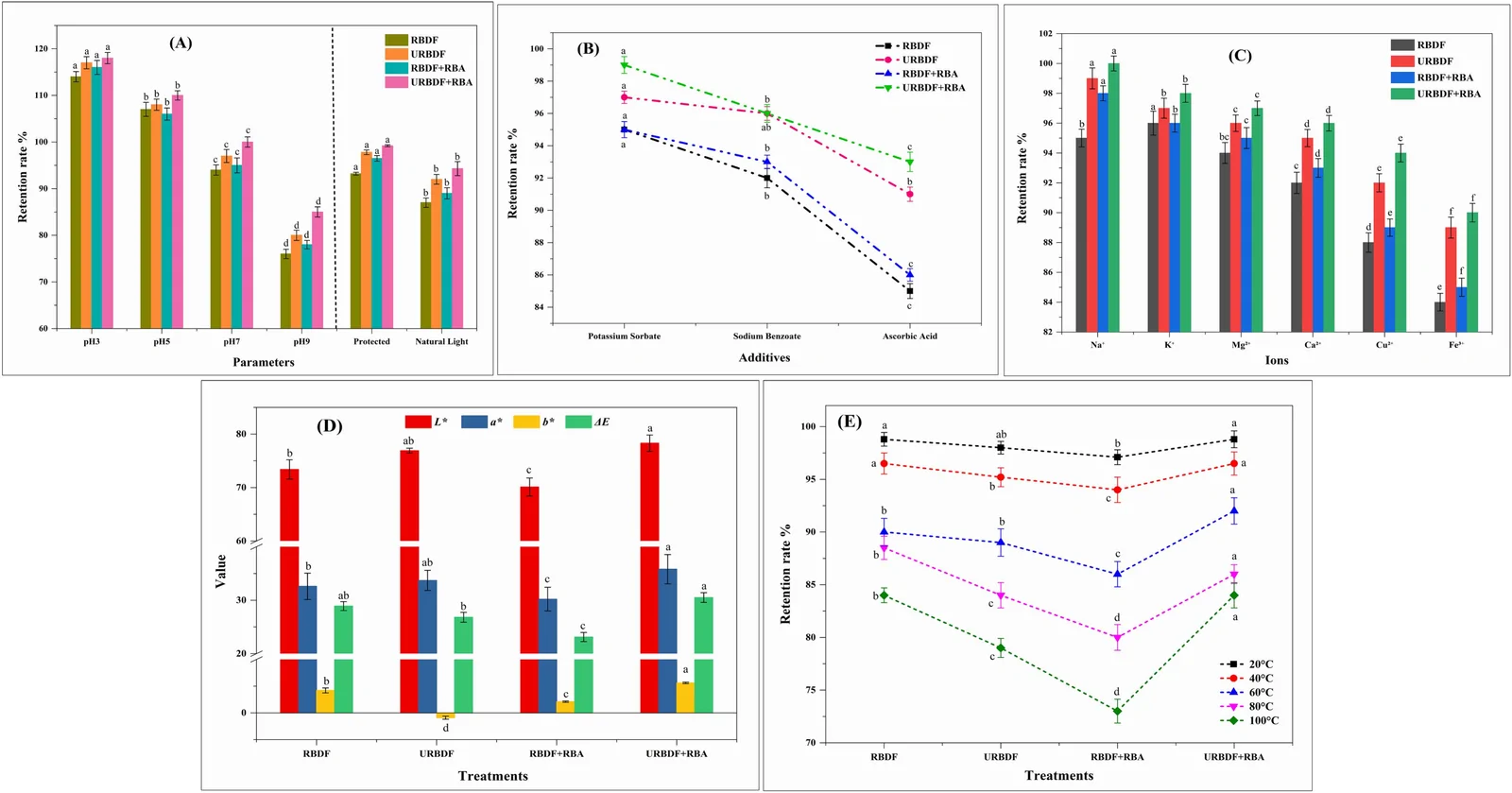
Stability studies (A) pH and light effects on RBDF, RBDF + RBA, URBDF and URBDF + RBA, (B) Additives effect on RBDF, RBDF + RBA, URBDF and URBDF + RBA, (C) Ions effect on RBDF, RBDF + RBA, URBDF and URBDF + RBA, (D) Color characteristics RBDF, RBDF + RBA, URBDF and URBDF + RBA, (E) Temperature effects on RBDF, RBDF + RBA, URBDF and URBDF + RBA.

This enhanced stability is attributed to electrostatic interactions, hydrogen bonding, and π–π stacking between RBDF and anthocyanins [46]. Ultrasonic cavitation creates a higher-surface-area, porous fiber network, enabling stronger interaction with anthocyanins. Entrapment of anthocyanins within micro-voids reduces light-induced oxidation. Similar observations were reported for soybean fiber malvidin complexes, confirming a universal stabilizing effect of insoluble dietary fiber on anthocyanins.

Temperature is a major driver of anthocyanin degradation, as shown in (Fig. 4E), where anthocyanin retention decreased with increasing temperature. After a 100-min water bath: At 20 °C, retention remained > 98 %, and at 80 °C, retention decreased by 11.37 % for RBA, 10.33 % for RBDF + RBA, and 79.2 % for URBDF + RBA at 100 °C. All treatments had < 89 % retention, but URBDF + RBA still performed best. Anthocyanin degradation at high temperature is primarily due to glycosidic bond cleavage and conversion to chalcone structures. The superior thermal stability of URBDF + RBA may result from the strengthened fiber network and improved microencapsulation produced by ultrasonic restructuring, which reduces heat-induced diffusion and oxidation. The improved stability can be explained by the fiber matrix acting as a physical barrier, hydrogen bonding preventing rapid deprotonation and hydrophobic interactions inhibiting anthocyanin structural rearrangement [47]. During ultrasound-induced fiber fibrillation, which increases binding sites and enhances protection at high temperatures, anthocyanins undergo hydrolysis of glycosides and formation of chalcone intermediates [48], [49]. Ring opening and fragmentation, however, were limited by restricting molecular mobility and shielding anthocyanins.

Anthocyanin stability is strongly pH-dependent (Fig. 4A) at pH ≤ 3 and retention remained > 90 %. At pH = 5, retention decreased: 25.53 % in RBA, 14.20 % in RBDF + RBA, 9.10 % in URBDF + RBA, and at pH ≥ 7, retention fell below 45 %. The protective effect at higher pH is attributed to dietary fiber shielding anthocyanins from rapid structural transformation into carbinol pseudo base and quinonoidal forms. URBDF especially slows this transition, likely due to stronger molecular interactions formed during ultrasonic modification [50]. These trends correspond to structural transitions: pH-dominant form stability, 1–2 flavylium cation (red). Very high, 4–5, carbinol pseudo base (colorless); moderate 6; quinoidal base (blue) Low ≥ deprotonated, degraded species. Very low URBDF + RBA showed significantly slower degradation due to tighter binding via ultrasound-enhanced microstructure, reduced hydration and lower exposure to an alkaline environment for the stabilization of the flavylium cation through fiber interactions [51]. This confirms that ultrasound-modified fiber improves structural stability across pH conditions.

Metal ions showed varying effects on anthocyanin stability (Fig. 4C): Na^+^, K^+^, Ca^2+^: no significant effect; Cu^2+^, Mg^2+^, Fe^3+^: caused substantial degradation. URBDF + RBA consistently showed the highest retention after 24 h. Anthocyanins readily form metal complexes that accelerate degradation; however, fiber adsorption reduces these interactions by physically embedding RBA and limiting direct metal contact [52].

Food preservatives (potassium sorbate, sodium benzoate, ascorbic acid) reduced anthocyanin stability (Fig. 4B). Ascorbic acid caused the greatest degradation, consistent with known ascorbic-anthocyanin condensation pathways [53]. Despite these degradative effects, URBDF + RBA still showed the highest retention, confirming that ultrasonic treatment greatly improves the protective efficiency of the fiber matrix.

### 3.12 Color characteristics

Changes in lightness (L*): As shown in (Fig. 4D), significant differences (p < 0.05) in L* values were observed among treatments, indicating that both ultrasonic treatment and anthocyanin adsorption markedly influenced the optical properties of red rice bran dietary fiber. RBDF exhibited a moderate L* value, reflecting the inherent brownish red pigmentation of red rice bran. After ultrasonic treatment, URBDF showed a significant increase in L*, suggesting that ultrasound disrupted the compact fiber matrix, reduced particle aggregation, and increased light scattering due to a more porous and irregular surface. Similar ultrasound-induced lightening effects have been reported for plant-based fibers and polysaccharides, attributed to cavitation-driven structural loosening and reduced chromophore shielding [54]. Upon complexation with red rice bran anthocyanins, RBDF + RBA displayed a significant decrease in L* compared with RBDF and URBDF, indicating effective anthocyanin adsorption and surface coating of the fiber. Anthocyanins, as strongly colored polyphenols, reduce sample brightness by absorbing visible light. Notably, URBDF + RBA exhibited the lowest L* value among all treatments, demonstrating that ultrasonic pretreatment enhanced anthocyanin loading capacity and color retention on the fiber matrix. This result is consistent with SEM and adsorption data, confirming that the increased specific surface area and exposed binding sites of URBDF promoted stronger pigment-fiber interactions.

Red-green coordinate (a*): The a* values, which reflect redness intensity, followed the order:

URBDF + RBA > RBDF + RBA > URBDF > RBDF. The relatively low a* value of RBDF indicates limited inherent redness after milling and processing. Ultrasonic treatment alone slightly increased a*, possibly due to exposure of internal phenolic residues embedded in the fiber matrix [55]. After anthocyanin adsorption, a* values increased significantly (p < 0.05), confirming successful incorporation of red rice bran anthocyanins into the fiber structure. Importantly, URBDF + RBA showed the highest a* value, indicating the strongest red coloration. This enhancement can be attributed to increased adsorption capacity of URBDF, Non-covalent interactions (hydrogen bonding and hydrophobic interactions) between anthocyanins and fiber and reduced anthocyanin degradation due to physical protection by the porous fiber network. These results demonstrate that ultrasound pretreatment not only improves adsorption efficiency but also preserves the chromatic intensity of anthocyanins. Yellow blue coordinate (b*): The b* values exhibited significant variation among treatments. RBDF showed a positive b* value, reflecting the presence of yellowish components such as residual lipids and Maillard-derived compounds. URBDF exhibited a reduced b* value, suggesting that ultrasonic cavitation partially removed or redistributed yellow-colored impurities and altered light reflection behavior. After anthocyanin binding, RBDF + RBA showed a further decrease in b*, consistent with masking of yellow tones by red and purple anthocyanins. Interestingly, URBDF + RBA showed a slight increase in b* compared with RBDF + RBA, which may be attributed to co-pigmentation effects and structural rearrangement of anthocyanins on the fiber surface. Such effects have been reported in anthocyanin polysaccharide systems, where molecular stacking and microenvironment polarity influence perceived hue.

Total color difference (*ΔE*): *ΔE* values provide an integrated assessment of overall color change compared with RBDF. All treated samples exhibited significantly higher *ΔE* values (p < 0.05), indicating that both ultrasonic treatment and anthocyanin adsorption substantially altered visual appearance [56]. Among all treatments, URBDF + RBA exhibited the highest *ΔE*, demonstrating the most pronounced color modification. This result confirms that ultrasonic pretreatment intensified anthocyanin binding and color expression, leading to a visually distinct and stable pigment-fiber complex. From an application perspective, a higher *ΔE* suggests greater potential for URBDF + RBA as a natural color carrier, functional food ingredient, or anthocyanin delivery matrix, particularly in systems where color stability and intensity are critical.

Mechanistic implications and practical significance: The results strongly support the structural and adsorption findings. Ultrasonic treatment enhanced fiber porosity and surface activity, facilitating anthocyanin immobilization and stabilizing chromatic properties [57]. The URBDF + RBA complex exhibited greater redness, reduced lightness, and maximal total color difference, confirming it as the optimal treatment among all samples. These findings demonstrate that ultrasound-assisted modification of red rice bran dietary fiber is an effective strategy for improving anthocyanin adsorption efficiency. Enhancing color intensity and stability, developing value-added functional ingredients from red rice bran by-products.

URBDF + RBA represents the best formulation, exhibiting the strongest anthocyanin binding, highest redness (a*), lowest lightness (L*), and greatest overall color difference (*ΔE*), highlighting the synergistic effect of ultrasonic treatment and fiber + anthocyanin complexation.

### 3.13 Antioxidant activity

The antioxidant properties of RBDF and its complexes were evaluated using DPPH, hydroxyl radical, and ABTS assays (Fig. 5A). RBDF alone exhibited moderate activity due to intrinsic phenolic residues. Ultrasonic treatment slightly enhanced activity by releasing bound antioxidants from the fiber matrix. RBDF + RBA complexes showed significantly improved scavenging abilities due to the addition of anthocyanins.

**Fig. 5:**
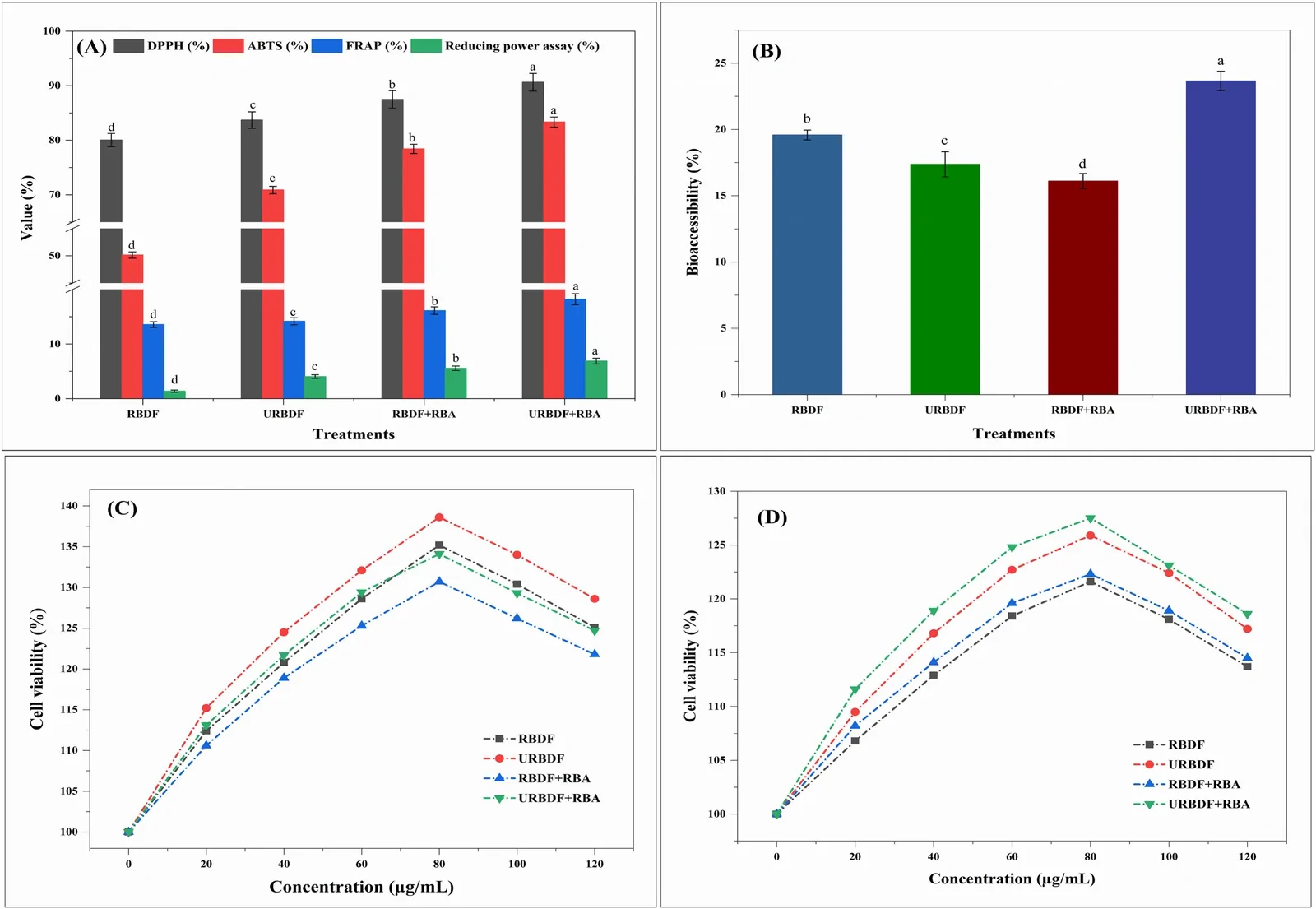
(A) DPPH, ABTS, FRAP and reducing power assay of RBDF, RBDF + RBA, URBDF and URBDF + RBA, (B) Bioaccessibility of RBDF, RBDF + RBA, URBDF and URBDF + RBA, (C) Cell proliferation activities on RAW 264.7 of RBDF, RBDF + RBA, URBDF and URBDF + RBA, and (D) LO2 cells proliferation activities against RBDF, RBDF + RBA, URBDF and URBDF + RBA.

URBDF + RBA exhibited the strongest antioxidant activity: DPPH: 86.73 %, Hydroxyl: 81.19 % and ABTS: 76.67 %. Ultrasound promotes stronger non-covalent interactions and deeper embedding of RBA within URBDF, reducing anthocyanin degradation and maximizing antioxidant retention [58]. This enhancement is attributed to: Non-covalent binding stabilizing anthocyanins against oxidation and to synergistic effects between dietary fiber phenolics and RBA. Ultrasonic fibrillation increases the loading and accessibility of anthocyanins [42]. These findings demonstrate that URBDF + RBA forms a robust antioxidant complex with high radical scavenging efficiency.

### 3.14 Cellular viability response of RAW 264.7 macrophages

Red rice bran dietary fiber (RBDF), ultrasound-treated red rice bran (URBDF), RBDF combined with red rice bran anthocyanins (RBDF + RBA), and URBDF + RBA significantly enhanced the cellular viability and metabolic activity of RAW 264.7 macrophages in a concentration-dependent manner, as assessed by the CCK-8 assay (Fig. 5C). All treatments increased cell viability within the tested concentration range (20–120 μg/mL), with the most pronounced effect observed at 60–80 μg/mL. Among the treatments, URBDF exhibited consistently higher cell viability than native RBDF, which may be associated with the improved physicochemical properties and increased accessibility of functional groups generated by ultrasound treatment.

Notably, the URBDF + RBA complex showed the greatest increase in cell viability, with viability reaching approximately 135–140 % at 80 μg/mL. This suggests a synergistic interaction between ultrasound-modified fiber and anthocyanins, increasing surface roughness, porosity, and specific surface area, facilitating non-covalent interactions, improved stabilization, and controlled release. The observed increase in CCK-8 signal indicates improved cellular metabolic activity and viability under the tested conditions. However, because the CCK-8 assay measures metabolic activity rather than specific immune functions, these results cannot distinguish among enhanced proliferation, cytoprotection, increased metabolic activity, or macrophage activation. Therefore, the present findings demonstrate favorable cellular compatibility. Potential mechanisms involving antioxidant-mediated cytoprotection or signaling pathways such as Nrf2/HO-1 and TLR4/NF-κB remain hypothetical and require confirmation using dedicated assays, including ROS determination, cytokine analysis, Western blotting, and pathway inhibition experiments. The ultrasound-induced disruption of the fiber matrix increases surface area, exposes functional polysaccharide epitopes, and may plausibly improve interactions between the fiber matrix and the cellular surface [59]. Previous studies have reported that structurally modified dietary fibers can influence macrophage-associated biological responses [60], [61]. However, because the present study evaluated cellular viability using the CCK-8 assay, these results demonstrate improved cellular compatibility. The present findings are consistent with a structure–activity relationship associated with ultrasound modification, but confirmation of the underlying biological mechanisms will require dedicated functional assays, including cytokine profiling, ROS determination, and pathway-specific molecular analyses.

Cytoprotective effects on L-02 normal hepatocytes: In contrast to RAW 264.7 cells, L-02 human normal hepatocytes exhibited moderate but stable responses following treatment with RBDF, URBDF, and anthocyanin complexes (Fig. 5D). All samples increased L-02 cell viability at low to moderate concentrations, with maximal effects observed at 60–80 μg/mL, after which a slight decline was detected at higher concentrations [62]. Among all treatments, URBDF + RBA again showed the highest cell viability, reaching approximately 125–128 %, indicating good hepatocyte compatibility and suggesting potential cytoprotective effects that require further mechanistic validation. Interestingly, the enhancement observed in L-02 cells was less pronounced than that in RAW 264.7 cells, reflecting the distinct biological roles of immune cells and hepatocytes. Similar discrepancies between macrophage and hepatocyte responses to anthocyanin have been widely reported [63].

The observed differences may be partially explained by fiber, anthocyanin composition, and molecular weight distribution. Previous studies have suggested that the stability and bioactivity of anthocyanins can vary based on their source and processing conditions, as seen in studies comparing black rice bran and purple hulls [64]. At the same time, lower molecular weight polysaccharides often show improved hepatoprotective activity due to enhanced cellular uptake [65]. This improved its solubility, thereby facilitating interaction with L-02 cells and enhancing proliferative activity.

Role of molecular weight and structural characteristics: The relationship between fiber, anthocyanin molecular weight and biological activity remains complex and system-dependent. While some studies have reported that lower molecular weight fractions exhibit stronger biological activity, others have shown that higher molecular weight fractions may exert superior effects depending on structural features [66]. The present results suggest that ultrasound-modified RBDF with reduced molecular weight displays enhanced cellular activity in L-02 cells, whereas the cellular response observed in RAW 264.7 macrophages appears more sensitive to the overall structural characteristics of the fiber and its interaction with anthocyanins. Furthermore, the formation of RBDF + anthocyanin complexes may stabilize anthocyanins and prolong their cellular action, leading to sustained antioxidant and cytoprotective effects. Similar synergistic effects have been observed in polysaccharide polyphenol systems, where complex formation improves bioaccessibility and functional performance [67].

Comparative cellular response and functional implications. Taken together, RAW 264.7 macrophages exhibited higher sensitivity to RBDF-based treatments than L-02 cells, demonstrating greater cellular responsiveness under the present experimental conditions. In contrast, the moderate yet consistent increases in L-02 cell viability indicate good cellular compatibility with the tested formulations. The superior performance of URBDF + RBA across both cell models underscores the importance of ultrasound modification and anthocyanin complexation in enhancing the bioactivity of dietary fiber. These findings support the potential application of ultrasound-treated red rice bran dietary fiber–anthocyanin complexes as multifunctional food ingredients with favorable cellular compatibility and potential health-promoting properties.

### 3.15 Bioaccessibility

Ultrasound-modified fiber enhances anthocyanin release and protection during digestion. Bioaccessibility, defined as the fraction of bioactive compounds released into the digestive fluids of the small intestine and thus available for absorption, is a critical indicator of functional efficacy in dietary polyphenols [68]. The in vitro gastrointestinal digestion assay (Fig. 5B) revealed pronounced differences in anthocyanin bioaccessibility across the four sample groups. Following sequential simulated gastrointestinal digestion, the bioaccessible anthocyanin fractions for RBA (free anthocyanin), RBDF + RBA, URBDF (unloaded), and URBDF + RBA were 19.4 %, 16.11 %, 17.37 %, and 23.66 % (p < 0.05), respectively. It is important to note that the lower bioaccessibility of RBDF + RBA relative to free RBA reflects the strong adsorptive binding of anthocyanins within the unmodified fiber matrix, which restricts their release during gastric and intestinal digestion. This is consistent with the well-documented trade-off between protection and accessibility in insoluble dietary fiber systems: unmodified RBDF forms a tight physicochemical barrier that shields polyphenols from degradation but simultaneously limits their immediate release.

In contrast, ultrasound-modified URBDF + RBA achieved the highest bioaccessibility (23.66 %), substantially exceeding that of free RBA (19.4 %). This superior performance is attributed to the porous microstructure formed by ultrasonic cavitation, which generates controlled release channels that enable anthocyanin molecules to be progressively released under the pH gradient of gastrointestinal transit. Moreover, the reduced particle size and increased surface area of URBDF enhance dispersibility and enzyme accessibility, enabling more efficient anthocyanin release in the intestinal phase. Collectively, these results demonstrate that ultrasound modification transforms RBDF from a simple physical sequestrant into a smart carrier capable of both protecting anthocyanins from degradation and promoting their controlled intestinal release. The improvement in anthocyanin bioaccessibility can be attributed to the formation of non-covalent interactions, predominantly hydrogen bonding and hydrophobic forces between anthocyanins and the polysaccharide matrix [47]. These interactions create a protective microenvironment that shields anthocyanins from degradation by pH fluctuations and digestive enzymes. The porous network-like structure of ultrasound-treated fiber encapsulates anthocyanins, functioning as a natural carrier that moderates release during gastrointestinal transit [69].

Furthermore, the decreased particle size and increased surface area of URBDF enhance its dispersibility and affinity toward anthocyanin molecules, promoting a stable complex formation. This mechanism mirrors previous findings where encapsulation within dietary fiber matrices, such as ethyl cellulose or gum Arabic, significantly improved polyphenol stability and absorption efficiency during digestion [70]. Therefore, ultrasound-assisted modification of red rice bran fiber not only improves anthocyanin retention during digestion but also potentially facilitates their release in the colon, where they may exert prebiotic and antioxidant effects contributing to gut and systemic health benefits.

## 4 Conclusion

This study demonstrates that ultrasound-assisted modification of red rice bran dietary fiber (RBDF) substantially enhances its capacity to form stable, functional complexes with red rice bran anthocyanins (RBA). Ultrasonic cavitation disrupted the fiber's compact cellulose-hemicellulose-lignin architecture, exposing additional hydroxyl and aromatic binding sites and increasing anthocyanin adsorption capacity; the resulting URBDF + RBA complexes were stabilized primarily through hydrogen bonding, π-π stacking, and hydrophobic interactions, which conferred markedly improved anthocyanin retention under thermal, light, pH, metal-ion, and additive stress, along with enhanced antioxidant activity and water- and oil-holding capacity relative to unmodified controls. *In vitro* digestion showed that URBDF + RBA achieved the highest bioaccessibility among all groups, reflecting controlled release through the fiber's porous channels rather than protection alone. In addition, preliminary CCK-8-based cell viability assays demonstrated favorable cellular compatibility in RAW 264.7 macrophages and L-02 hepatocytes under the tested conditions. Novelty: This work is the first to systematically characterize the binding thermodynamics and multi-site interaction mechanism between ultrasound-modified red rice bran dietary fiber and its co-located cyanidin-3-glucoside-rich anthocyanins, demonstrating that ultrasound treatment simultaneously improves protective capacity and controlled intestinal release, resolving a key functional trade-off in fiber-polyphenol carrier design and offering a practical strategy for valorizing red rice bran by-products into stable, bioaccessible anthocyanin delivery systems for functional foods. Limitations and future directions: This study relied on static in vitro digestion and single-cell-type assays, with binding constants estimated from UV–Vis data; future work should incorporate dynamic gut models and in vivo studies to validate bioaccessibility, isothermal titration calorimetry and molecular docking for precise thermodynamic characterization, and cytokine profiling, ROS determination, and NF-κB pathway analysis to investigate whether immunomodulatory mechanisms contribute to the observed cellular responses. Scale-up feasibility and stability under industrial food processing (e.g., extrusion, spray drying) also warrant evaluation.

## CRediT authorship contribution statement

**Zahoor Ahmed:** Writing – review & editing, Writing – original draft, Software, Investigation, Formal analysis, Conceptualization. **Muhammad Faisal Manzoor:** Writing – review & editing, Writing – original draft, Software, Investigation. **Umar Farooq:** Writing – review & editing, Software. **Yibin Zhou:** Writing – review & editing, Supervision, Resources, Project administration, Funding acquisition. **Abderrahmane Aït-Kaddour:** Writing – review & editing. **Tao Sheng:** Writing – review & editing, Software. **Chen Chen:** Writing – review & editing, Software. **Rana Muhammad Aadil:** Writing – review & editing. **Marwa Ezz El-Din Ibrahim:** Writing – review & editing.

## Funding

This work was supported by the Deanship of Scientific Research, Vice Presidency for Graduate Studies and Scientific Research, King Faisal University, Saudi Arabia [GRANT: KFU261319].

## Declaration of competing interest

The authors declare that they have no known competing financial interests or personal relationships that could have appeared to influence the work reported in this paper.

## Appendix A Supplementary data

Supplementary Data 1 Supplementary material indicates the response surface methodology for process optimization.
