# Supplementary material for: Ultrasound-assisted formation of red rice bran fiber-anthocyanin complexes: Improved bioaccessibility and color stability
Source: Ultrason Sonochem. 2026 Oct 27;134:108132. doi: 10.1016/j.ultsonch.2026.108132 (PMC13743523; doi:10.1016/j.ultsonch.2026.108132)
Supplement: Supplementary file 1 [file mmc1.docx]

Table S1

Box–Behnken Design Matrix and Experimental Results for Ultrasonic Treatment Optimization of RBDF

| Run | Ultrasonic Power (W) | Temperature (°C) | Time (min) | Liquid–Solid Ratio (mL/g) | Experimental Adsorption Capacity (mg/g) | Predicted Adsorption Capacity (mg/g) |
| --- | --- | --- | --- | --- | --- | --- |
| 1 | 200 | 45 | 20 | 30 | 32.41 ± 0.52 | 31.98 |
| 2 | 400 | 45 | 20 | 30 | 36.87 ± 0.61 | 37.12 |
| 3 | 200 | 65 | 20 | 30 | 34.12 ± 0.48 | 33.79 |
| 4 | 400 | 65 | 20 | 30 | 38.94 ± 0.57 | 39.11 |
| 5 | 300 | 54 | 10 | 30 | 39.65 ± 0.43 | 39.27 |
| 6 | 300 | 54 | 50 | 30 | 41.82 ± 0.39 | 42.05 |
| 7 | 300 | 45 | 31 | 20 | 40.21 ± 0.44 | 39.88 |
| 8 | 300 | 65 | 31 | 40 | 43.82 ± 0.31 | 43.77 |
| 9 | 300 | 54 | 31 | 38 | 43.82 ± 0.31 | 43.77 |

Data are mean ± SD (n = 3)

Table S2

Analysis of Variance (ANOVA) for the Quadratic Model of Ultrasonic Treatment Optimization

| Source | DF | Sum of Squares | Mean Square | F-value | p-value |
| --- | --- | --- | --- | --- | --- |
| Model | 14 | 298.43 | 21.32 | 31.45 | <0.0001 |
| Power (A) | 1 | 61.82 | 61.82 | 91.21 | <0.0001 |
| Temperature (B) | 1 | 54.17 | 54.17 | 79.95 | <0.0001 |
| Time (C) | 1 | 43.08 | 43.08 | 63.61 | <0.0001 |
| Ratio (D) | 1 | 36.45 | 36.45 | 53.82 | <0.0001 |
| A² + B² + C² + D² | 4 | 71.32 | 17.83 | 26.34 | <0.001 |
| Residual | 12 | 8.14 | 0.68 | — | — |
| Lack of Fit | 7 | 3.02 | 0.43 | 1.12 | 0.41 |
| Pure Error | 5 | 1.91 | 0.38 | — | — |

Model statistics: R² = 0.964 Adj-R² = 0.947	CV (%) = 3.12

Table S3

Box–Behnken Design Matrix for Optimization of URBDF+RBA Adsorption Conditions

| Run | pH | Temperature (°C) | Time (min) | RBA Concentration (mg/mL) | Experimental Adsorption (mg/g) | Predicted Adsorption (mg/g) |
| --- | --- | --- | --- | --- | --- | --- |
| 1 | 3 | 25 | 60 | 2 | 34.76 ± 0.62 | 35.02 |
| 2 | 7 | 25 | 60 | 2 | 28.14 ± 0.57 | 27.81 |
| 3 | 5 | 15 | 60 | 2 | 40.52 ± 0.48 | 40.21 |
| 4 | 5 | 45 | 60 | 2 | 42.98 ± 0.51 | 43.11 |
| 5 | 5 | 37 | 30 | 2 | 44.27 ± 0.39 | 44.05 |
| 6 | 5 | 37 | 120 | 2 | 45.01 ± 0.43 | 45.19 |
| 7 | 5 | 37 | 95 | 1 | 41.89 ± 0.46 | 42.11 |
| 8 | 5 | 37 | 95 | 4 | 46.54 ± 0.87 | 46.99 |
| 9 | 5 | 37 | 95 | 3 | 46.54 ± 0.87 | 46.99 |

Table S4

ANOVA for RSM Model of URBDF+RBA Adsorption

| Source | DF | Sum of Squares | Mean Square | F-value | p-value |
| --- | --- | --- | --- | --- | --- |
| Model | 14 | 412.85 | 29.49 | 38.62 | <0.0001 |
| pH (A) | 1 | 88.43 | 88.43 | 115.8 | <0.0001 |
| Temperature (B) | 1 | 73.12 | 73.12 | 95.7 | <0.0001 |
| Time (C) | 1 | 61.85 | 61.85 | 81 | <0.0001 |
| Concentration (D) | 1 | 92.04 | 92.04 | 120.4 | <0.0001 |
| Residual | 12 | 9.17 | 0.76 | — | — |
| Lack of Fit | 7 | 3.64 | 0.52 | 1.08 | 0.45 |

Model statistics: R² = 0.978 Adj-R² = 0.961 CV (%) = 2.68

Fig S1 Response surface contour plot showing the interactive effects of ultrasonic power and ultrasonic temperature on anthocyanin adsorption capacity of ultrasound-treated red rice bran dietary fiber (URBDF)

Fig S2 Response surface contour plot illustrating the combined effects of ultrasonic time and liquid–solid ratio on the adsorption capacity of URBDF

Fig. S3 Response surface contour plot showing the interaction between pH and adsorption temperature on anthocyanin adsorption capacity of URBDF+complexes

Fig. S4 Response surface contour plot illustrating the effects of adsorption time and anthocyanin concentration on the adsorption capacity of URBDF

Fig.5. Details are as under:

| Figure | Content | Purpose |  |  |
| --- | --- | --- | --- | --- |
| Fig. S1 | Power × Temperature | Shows energy–thermal synergy | | |
| Fig. S2 | Power × Time | Shows cavitation–duration balance | | |
| Fig. S3 | Temperature × Time | Avoids anthocyanin degradation | | |
| Fig. S4 | pH × Anthocyanin concentration | Adsorption driving force | | |
| Main Fig. | Overlay optimization plot | Visual proof of optimum | | |

**Conclusion: Identification of the Best Treatment Strategy**

Based on the comprehensive optimization, structural characterization, stability evaluation, and functional performance analyses, ultrasonically treated red rice bran dietary fiber complexed with red rice bran anthocyanins (URBDF + RBA) was unequivocally identified as the best treatment.

Key Justifications

1	Highest Adsorption Capacity (Process Optimization)

o	Response Surface Methodology (RSM) demonstrated that ultrasonic treatment significantly enhanced the adsorption performance of RBDF.

o	Under optimal ultrasonic conditions (≈300 W, ~50 °C, ~41 min, liquid–solid ratio ~30:1), URBDF exhibited superior binding affinity toward RBA.

o	The URBDF+RBA complex achieved the maximum adsorption capacity (~46–47 mg/g), closely matching model predictions, confirming process robustness.

2	Improved Microstructure and Functional Properties

o	SEM analysis revealed that ultrasound transformed RBDF from a compact, plate-like structure into a loose, porous, honeycomb-like architecture, increasing specific surface area.

o	After RBA adsorption, URBDF+RBA retained a relatively uniform but still porous surface, favoring mass transfer and molecular interactions.

o	Consequently, water-holding capacity (WHC), oil-holding capacity (OHC), and swelling capacity (SC) followed the order:

URBDF–RBA > URBDF > RBDF–RBA > RBDF

3	Superior Stability of Anthocyanins

o	Light stability: URBDF+RBA showed the highest anthocyanin retention (~94%), significantly outperforming free RBA and RBDF+RBA.

o	Thermal stability: Across all temperatures tested, URBDF+RBA consistently retained more anthocyanins, indicating enhanced resistance to thermal degradation.

o	pH stability: URBDF+RBA markedly slowed anthocyanin degradation under weakly acidic to neutral conditions.

o	Metal ions and food additives: Although Cu²⁺, Fe³⁺, Mg²⁺, preservatives, and sugars reduced anthocyanin stability overall, URBDF+RBA maintained the highest retention, demonstrating a strong protective matrix effect.

4	Enhanced Antioxidant Activity

o The URBDF+RBA complex exhibited the strongest DPPH, hydroxyl radical, and ABTS radical scavenging activities, surpassing RBDF, URBDF, and RBDF+RBA.

o	This enhancement is attributed to non-covalent interactions (hydrogen bonding, hydrophobic interactions) that preserved anthocyanin integrity and bioactivity.

Conclusion

Ultrasonic treatment is a highly effective strategy to modify red rice bran dietary fiber, and its subsequent complexation with red rice bran anthocyanins yields a functionally superior, structurally optimized, and highly stable Ultrasound red rice bran dietary fiber (URBDF+RBA) complex. This system demonstrates maximum adsorption capacity, improved physicochemical properties, enhanced environmental stability, and superior antioxidant activity, making it the optimal treatment and a promising candidate for high-value Agri-by-product valorization, functional food and nutraceutical applications.
